# Viral biogeography of the mammalian gut and parenchymal organs

**DOI:** 10.1038/s41564-022-01178-w

**Published:** 2022-08-02

**Authors:** Andrey N. Shkoporov, Stephen R. Stockdale, Aonghus Lavelle, Ivanela Kondova, Cara Heuston, Aditya Upadrasta, Ekaterina V. Khokhlova, Imme van der Kamp, Boudewijn Ouwerling, Lorraine A. Draper, Jan A.M. Langermans, R Paul Ross, Colin Hill

**Affiliations:** 1 APC Microbiome Ireland, University College Cork, Cork, Ireland; 2 School of Microbiology, University College Cork, Cork, Ireland; 3 Biomedical Primate Research Centre, Rijswijk, The Netherlands; 4 Department of Population Health Sciences, Veterinary Faculty, Utrecht University, Utrecht, The Netherlands

## Abstract

The mammalian virome has been linked to health and disease but our understanding of how it is structured along the longitudinal axis of the mammalian gastrointestinal tract (GIT) and other organs is limited. Here we report a metagenomic analysis of the prokaryotic and eukaryotic virome occupying luminal and mucosa-associated habitats along the GIT, as well as parenchymal organs (liver, lung and spleen), in two representative mammalian species, the domestic pig and rhesus macaque (six animals per species). Luminal samples from the large intestine of both mammals harboured the highest loads and diversity of bacteriophages (class *Caudoviricetes*, family *Microviridae* and others). Mucosal samples contained much lower viral loads but a higher proportion of eukaryotic viruses (families *Astroviridae*, *Caliciviridae*, *Parvoviridae*). Parenchymal organs contained significant numbers of bacteriophages of gut origin, in addition to some eukaryotic viruses. Overall, GIT virome composition was specific to anatomical region and host species. Upper GIT and mucosa-specific viruses were greatly under-represented in distal colon samples (a proxy for faeces). Nonetheless, certain viral and phage species were found ubiquitously in all samples from the oral cavity to the distal colon. The dataset and its accompanying methodology may provide an important resource for future work investigating the biogeography of the mammalian gut virome.

## Introduction

The gastrointestinal tracts (GIT) of humans and other mammals contain highly individualized microbiomes^1–4^, composed of bacteria^5^, archaea^6^, eukaryotic microorganisms^7^, and viruses^8–10^. The close association of microbes and their mammalian host as an ecological unit is increasingly recognised as important for health^11,12^. The gut virome, which is largely composed of bacterial viruses (bacteriophages or phages) remained a relatively unexplored area until recently, when a potential role for the virome in shaping bacterial communities was postulated^13–16^. A number of potential mechanisms by which such shaping could occur have been suggested, and include “kill-the-winner” dynamics in bacterial communities caused by phage predation (at least at strain and sub-strain level^17^); diversifying selection acting upon both adaptive mutations^18,19^ and phase variations^20,21^; as well as phage-mediated horizontal gene transfer (HGT) that could involve diverse mechanisms such as generalised, specialised and lateral transduction^22–24^.

Our current understanding of the virome, and the phageome in particular, is limited and based mostly on sequencing-based studies of faecal samples, which represent static snapshots of the distal gut virome. Neither the temporal dynamics^17^, nor the variation and flux of viral populations along the longitudinal and transverse axes of GIT (the “viral biogeography” of the gut^25,26^), have received proper attention. Recent human cohort studies highlighted a tight association between the gut virome and gut bacteriome in terms of both α- and β-diversity^17,27^. Additionally, multiple lines of evidence suggest that many successful gut bacteriophages, such as the crAss-like phages, engage in long-term persistent relationships with their hosts^28,29^, in line with the “piggyback-the-winner” dynamics of temperate bacteriophages^30^. It is important to obtain a more detailed view of both the temporal and spatial dynamics of the virome in order to understand its interplay with the bacterial microbiome, its significance for human health and potential role in disease^31,32^.

Complex macro- and micro-anatomy of the digestive tract, together with exocrine functions of GIT mucosa and accessory organs create a series of longitudinal and radial biochemical gradients, affecting the composition of local resident microbiota, including viruses^25,33,34^. Adaptation to such microhabitats is clearly evident amongst bacteria, such as body site-specific lactobacilli^35^ or various mucin-foraging bacteria^36^. Host-associated mutualistic and commensal bacteria have evolved persistence mechanisms such as adsorption and embedding into mucus layers, and potentially have access to anatomical sites protected from the luminal stream and the action of bacteriophages^25,34^. Similarly, the ability to bind to and accumulate in the mucous layer and potentially restrict bacterial invasion was also reported for certain bacteriophages, which prompted a discussion on the role of bacteriophages as a quasi-immune system of the digestive tract^37–39^. Pronounced physiological and anatomical differences between homologous GIT segments in different species of mammals, associated with digestive adaptations, adds another layer of complexity to this system^40^.

In this study, we present a comprehensive biogeographical analysis of viruses in the GIT of two mammalian species, the domestic pig (*Sus scrofa domesticus*) and rhesus macaque (*Macaca mulatta*), chosen for their phylogenetic, physiological and anatomical relevance for humans. We focuse our attention on bacteriophage populations and attempt to answer two key questions. Firstly, what are the differences in virome composition between different digestive tract regions and how representative are distal gut samples of the virome in the upper GIT? Secondly, to what extent can the virome be shared between the digestive tract regions and with extra-GIT organs?

## Results

### Virome sequencing approach

**Fig. 1:**
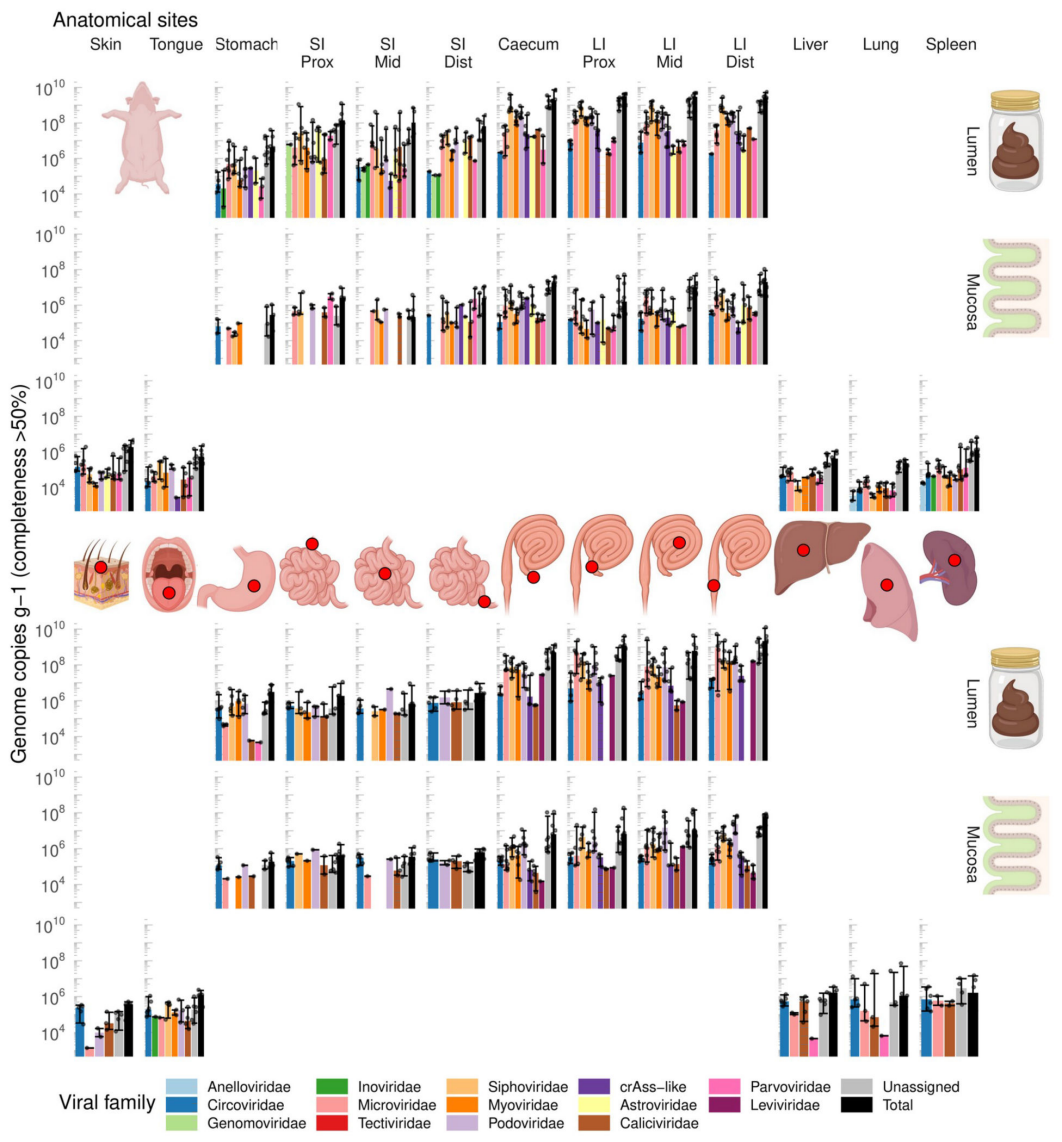
Abundance of different viral families along the GIT and in parenchymal organs of domestic pigs (A, n=6) and rhesus macaques (B, n=6). SI, small intestine; LI, large intestine; Prox/Mid/Dist, proximal, medial and distal portions, respectively. Absolute abundance of viral genomes was calculated by comparing coverage with that of the spike-in standard (phage Q33). Only genomes with >50% of estimated completeness were taken into account when calculating viral loads. Bar heights correspond to median values across six animals of each species, error bars denote interquartile ranges. Rows of plots in each of panel A and B are tissue types (top to bottom: lumen, mucosa, and skin/parenchyma). Columns of plots are anatomical sites. Middle portion of the figure shows schematic locations of sampled anatomical sites.

We applied shotgun metagenomic sequencing of VLP-enriched samples^41–43^, to characterize luminal/mucosal viral DNA and RNA content in different locations along the digestive tract. In order to adopt a broader taxonomic outlook and get insights into spatial virome organisation that go beyond the physiological and anatomical specifics of a particular mammalian species, we included six healthy domestic pigs (*Sus scrofa domesticus*) and six rhesus macaques (*Macaca mulatta*). Thirteen anatomical locations were sampled for each species, including skin, tongue, stomach, small intestine (SI; proximal [duodenum], medial [jejunum], and distal [ileum]), caecum, large intestine (LI; proximal, medial, and distal colon), as well as parenchymal organs (liver, lung and spleen). At relevant sites, both the mucosal and the surrounding luminal content were sampled (Fig. 1). Given the overwhelming prevalence of bacteriophages in mammalian faecal viromes^17,41^, their possible role in shaping the gut bacteriome^16,19,44^ and a lack of knowledge on their spatial distribution and populations dynamics in the GIT^9,34^, bacterial viruses were the primary focus of this study.

Genomic DNA and cDNA of mixed viral populations were sequenced using the Illumina Novaseq platform to a median depth of 6.2±5.8M per sample (median±IQR; Supplementary Information). Unlike many previous studies, our viral metagenomics approach was designed to be relatively unbiased. A simple nucleic acid extraction procedure was adopted that deliberately avoided the use of micro-filtration, VLP precipitation using PEG/NaCl, chloroform extraction, or density gradient ultracentrifugation; all of which are known to introduce different biases in virome profiling studies^42,43,45^. By avoiding whole-genome amplification we also avoided artificial virome composition skewness, loss of viral diversity, and over-amplification of small circular ssDNA genomes^17,46^. Lastly, including lactococcal phage Q33 as an artificial internal viral standard in our extraction procedure allowed us to estimate abundance of viral genomes in the sample by comparing their mean sequence coverage with that of the internal standard^17,43^.

**Extended Data Fig. 1:**
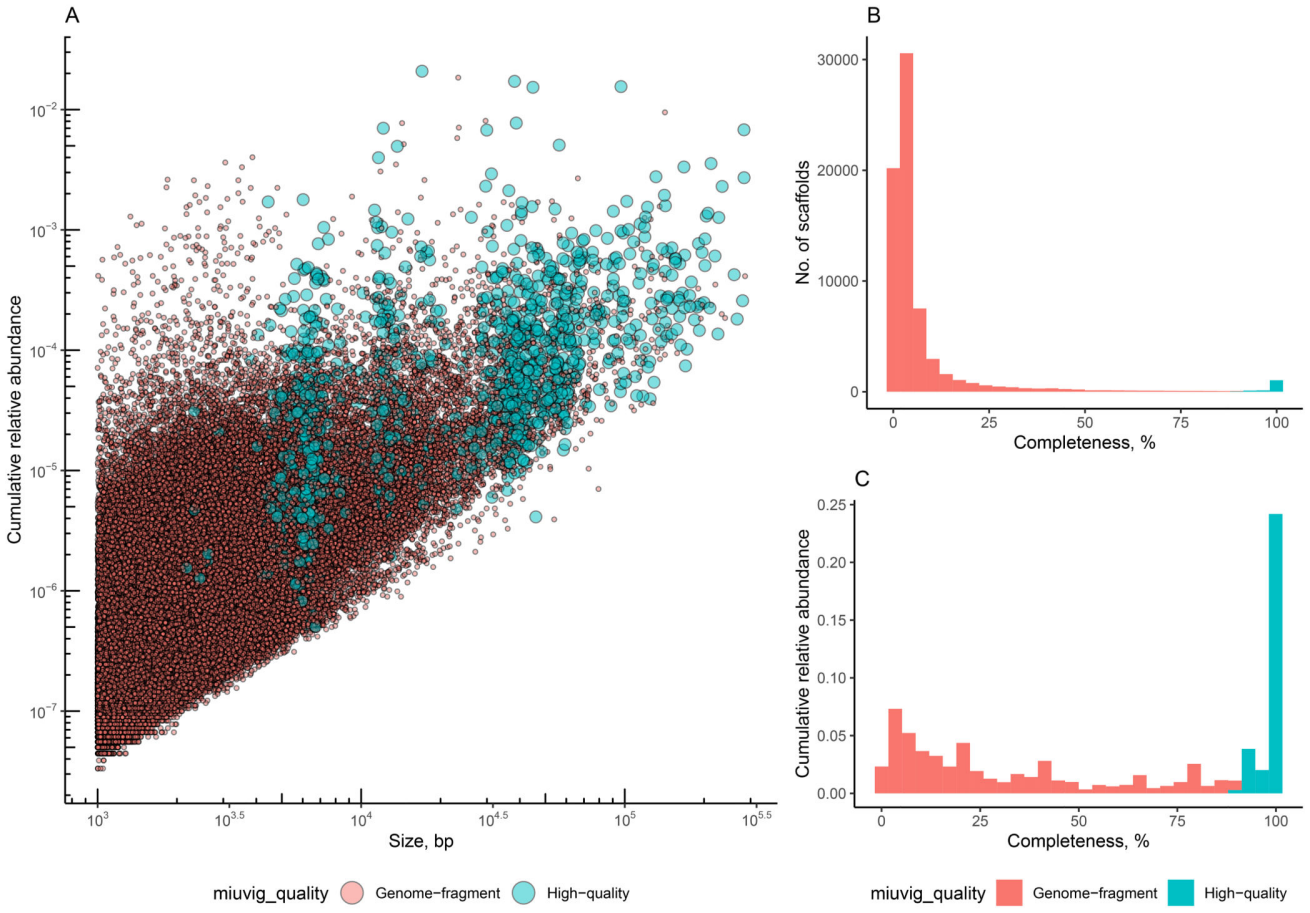
Catalogue of viral genomic contigs assembled from trimmed and filtered Illumina reads (n = 107,680). A, Average read coverage vs. contig length, categories of viral genomic contigs identified by CheckV (high-quality genomes vs genome fragments according to definitions given by the MIUViG standard); B, distribution of viral genomic contigs by completeness level as predicted by CheckV with high quality draft and complete genomes by MIUViG standard highlighted in blue; C, cumulative fractional abundance of genomic contigs with different levels of completeness.

**Extended Data Fig. 2:**
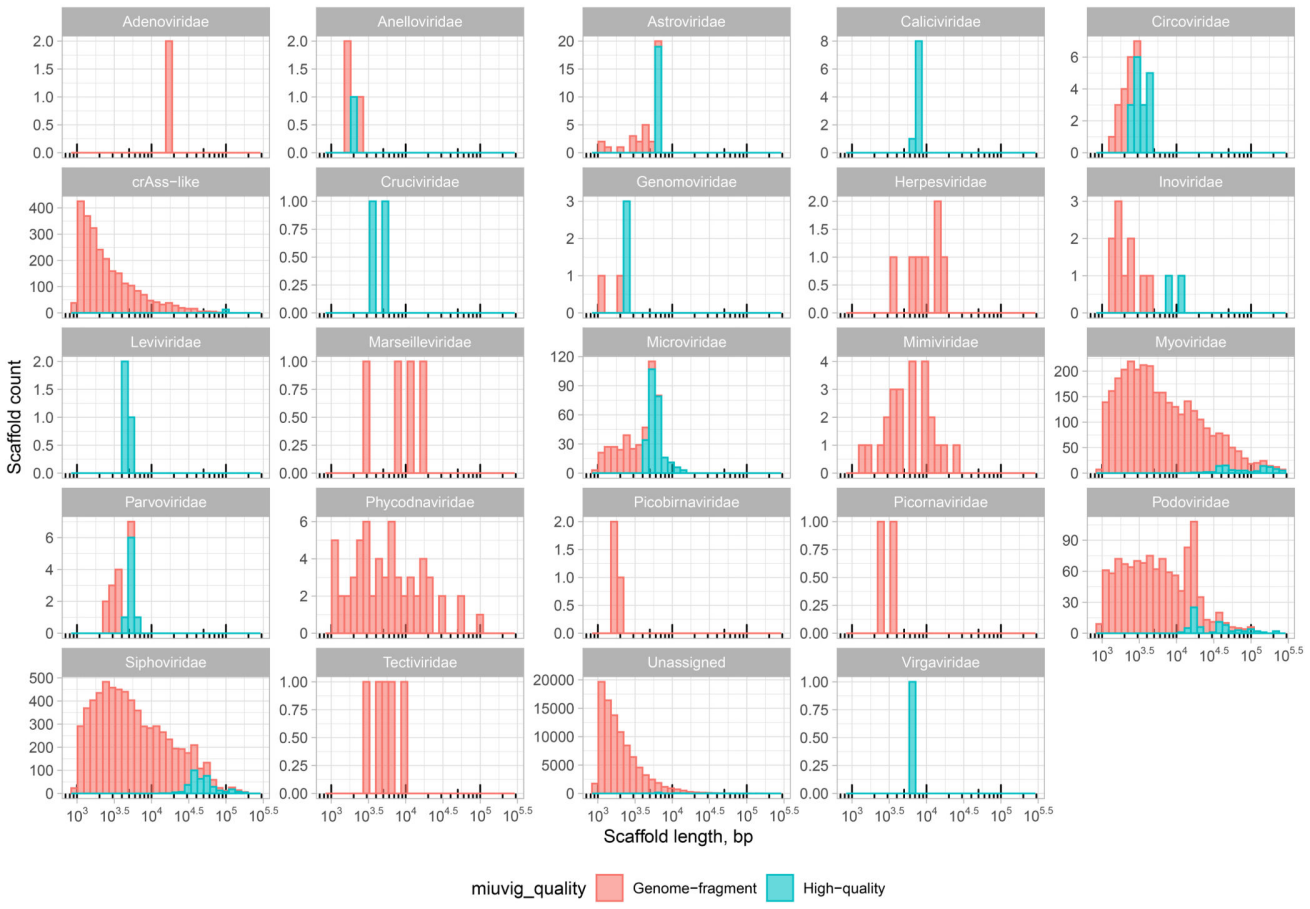
Taxonomic distribution, size, and completeness of viral genomic contigs. Different viral families are shown in separate panels. Assignments are based on Demovir script. Contig size is plotted on log10-scaled x-axis. Contig completeness is predicted using CheckV.

Assembly of reads into contigs^47^, removal of redundancy across individual samples and animals^17^, and selection of viral sequences from a bacterial and mammalian host DNA background yielded of catalogue of 107,680 contigs, corresponding to putative complete and fragmented viral genomes (Extended Data Fig. 1). At least 24 families of prokaryotic and eukaryotic viruses^48,49^ were recognised across the viromes of the two mammalian species using an automated taxonomic assignment algorithm (Extended Data Fig. 2). Approximately half (58,573) of the contigs were broadly similar (≥50% sequence identity over 85% of contig length) to previously reported genomes of either cultured or uncultured viruses^50–55^, but the remaining half were only identifiable as viral using a *de novo* multi-classifier approach^56^. However, even within sequences homologous to previously reported viral genomes and genome fragments, the majority (31,032) constitute unclassified viral species by the recently proposed standard of metagenomic viral species delineation (≥95% sequence identity over ≥85% of its length)^57^.

### Absolute viral counts along the GIT proximal-distal axis

Absolute quantitation of viral genomic contigs with ≥50% calculated completeness level (n = 2,442), grouped at viral family level, revealed pronounced differences in the virome between GIT locations, as well as the differences between the two animal species. The pig LI lumen is dominated by tailed bacteriophages (class *Caudoviricetes*, including crAss-like phages^9,53,58^) with total viral loads exceeding 10^9^ genome copies g^-1^ contents. Similar total counts are evident in macaques, although small ssDNA *Microviridae* phages^59^ are the most numerous group of taxonomically classified viruses (Fig. 1). Total viral loads in large intestinal mucosa samples were two orders of magnitude lower than matched luminal samples, and eukaryotic viruses (families *Circoviridae*, *Astroviridae*, *Calicivirdae* and *Parvoviridae*) had higher relative weights in those locations. Stomach and SI lumen and mucosa were colonised by relatively even mixes of bacteriophages and eukaryotic viruses, with a characteristic prevalence of *Parvoviridae* in the pig small intestinal mucosa. Similar combinations of viral families were detectable in tongue mucosa and skin samples in both animal species.

Interestingly, samples taken from lung, spleen and liver parenchyma in both species contained unexpectedly high viral loads, approaching and exceeding 10^6^ genome copies g^-1^ of tissue. In macaques, these viral populations that are apparently associated with interior body milieu of healthy animals, were mainly represented by eukaryotic viruses of *Circoviridae* and *Caliciviridae* families. In both species, and especially in pigs, the viral consortia of interior milieu included bacteriophages, primarily from the *Microviridae* family (Fig. 1).

**Fig. 2:**
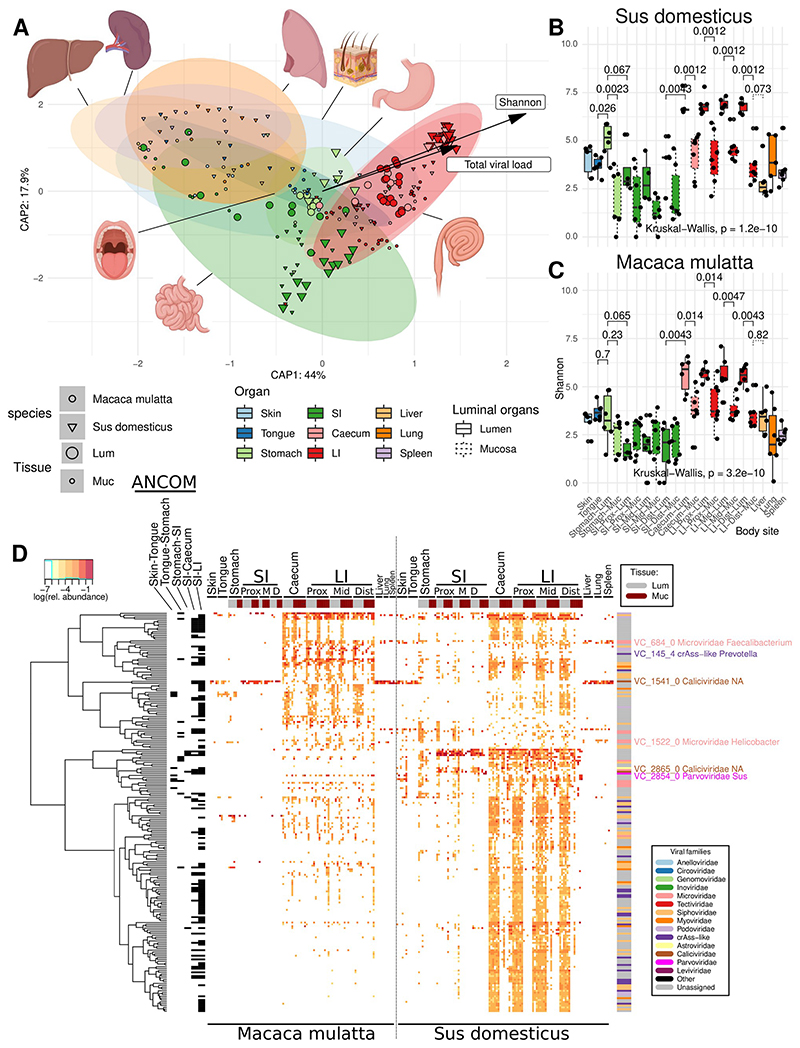
α- and β-Diversity of viromes in various anatomical sites in domestic pigs (n=6) and rhesus macaques (n=6). **A**, Canonical analysis of principal coordinates (CAPSCALE) of Bray-Curtis dissimilarities between virome samples, based on fractional VC counts; anatomical locations, Shannon diversity index and total viral load used as constraining explanatory variables (vectors are only shown for the latter two); ellipses represent 95% confidence regions (see colour legend below, common with panels B and C); **B** and **C**, Shannon diversity index calculated with read counts for individual viral genomic contigs (pigs and macaques, respectively); SI, small intestine; LI, large intestine; Prox/Mid/Dist, proximal, medial and distal portions, respectively; organ colours are matched with those in panel A, dashed boxplots represent mucosal sites; boxplots are standard Tukey type with interquartile range (box), median (bar) and Q1 – 1.5 × IQR/Q3 + 1.5 × IQR (whiskers); **D**, VCs differentially abundant between organs selected using ANCOM-II test (p < 0.05 after Benjamini-Hochberg correction); rows represent VCs, columns – sites in individual animals; a series of post-hoc tests identified VCs (annotated with black bricks) discriminatory between the following anatomic locations: Skin-Tongue, Tongue-Stomach, Stomach-SI, SI-Caecum, and SI-LI; the top and the right-hand side annotation bars represent tissue types (lumen vs mucosa) and viral families of VCs respectively; tree represents hierarchical clustering of VCs based on relative abundance patterns. An expanded version of this panel is provided as supplementary Fig. 5.

We then used all 107,680 viral contigs, both high quality and highly fragmented^57^, to identify compositional virome differences between different body sites in both animal species (Fig. 2; Extended Data Fig. 1). While highly fragmented viral contigs are less useful for taxonomic classification and host identification purposes^17,32^, omitting them from diversity analyses would leave the majority of viral diversity untapped (>50% of all Illumina reads from most body sites) (Supplementary Information). To compensate for inter-individual virome differences and make the virome more comparable across animal cohorts we used gene sharing networks^60^ to group all non-singleton viral genomic contigs (n = 12,262) into 3,770 Viral Clusters (VCs).

### Virome composition along the GIT proximal-distal axis

**Extended Data Fig. 3:**
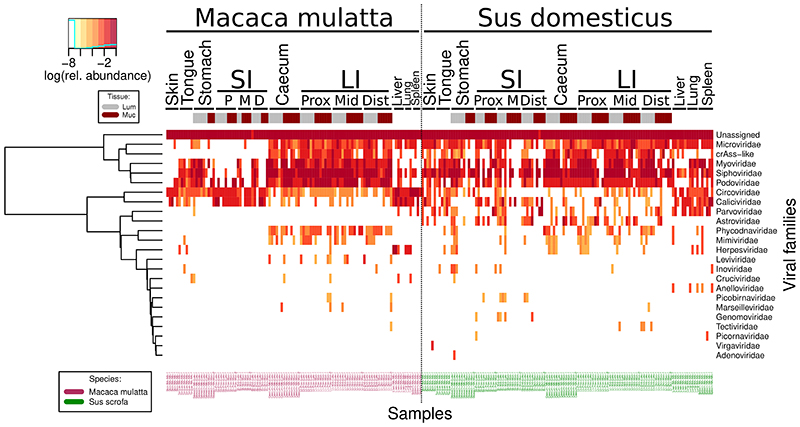
Aggregated fractional abundance of viral families across all anatomical sites in pigs (n = 6) and macaques (n = 6) in the study. Rows represent viral families, columns – sites in individual animals; the top annotation bar represent tissue types (lumen vs mucosa). Data is log10-transformed and presented with hierarchical clustering based on relative abundance patterns.

Multivariate virome comparison, based on fractional abundance of VCs at different sites, revealed a strong separation of large intestinal viromes from the small intestinal and gastric viromes in both animal species (Fig. 2A; Extended Data Fig. 3). When viewed across the two species, differences between organs were responsible for 11.1% of variance (Adonis with 1000 permutations, p ≤ 0.001). Surprisingly, inter-individual virome differences accounted for 9.6% of variance, higher than percent variance explained by animal species (4.9%; p ≤ 0.001). This is despite the fact that within each cohort animals were relatively inbred, lived in the same facility and were fed with a standardised diet. Moreover, between organ variance in interaction with the individual animal factor accounted for 30.0% of virome data variance (p ≤ 0.001), much higher than percent variance explained by similar interaction between organ and animal species factors (9.4%, p ≤ 0.001). Differences between mucosal and luminal virome explained only a relatively minor fraction of variance (1.0% for the main effect, 1.9% in interaction with organ factor; p ≤ 0.001). The major compositional separation between viromes of LI, SI and other organs seems to be closely aligned with overall diversity and total viral load (p ≤ 0.001 in PERMANOVA), with caecal and LI viromes being simultaneously the most taxonomically diverse and the most populous (Fig. 1; Fig. 2A-C).

In a single macaque (M6) and pig (E6), all mucosal sites were sampled twice, with 1 cm separation between each pair of samples, to assess whether close proximity of mucosal sites in the gut correlates with increased similarity of the virome composition. In both small and large intestine there was a tendency for these paired samples to resemble each other more closely than more distant sites within these and other animals, but this did not reach the level of statistical significance (see Supplementary Information).

We attempted to identify specific VCs driving the separation between organ-specific viromes (Fig. 2D), as well as VCs responsible for separation between luminal and mucosal viromes and the two animal species. Across the two species, a total of 217 VCs were differentially abundant between organ pairs in the following sequence: skin-tongue-stomach-SI-Caecum-LI (p < 0.05 in ANCOM test with Benjamini-Hochberg correction; see Supplemental results for more detail), with the largest fraction of these VCs (n = 119) being discriminatory between the SI and caecum/LI. Twenty VCs were found to be differentially abundant between luminal and mucosal sites in both species of animals, eleven of them being over-represented in mucosal sites compared to the luminal sites in the same organs. As described in the Supplemental results, many of the organ-discriminatory VCs were positively correlated with bacterial genera characteristic of a particular segment in the GIT (see Supplementary Information).

### Sharing of virome components between different regions in the GIT

**Extended Data Fig. 4:**
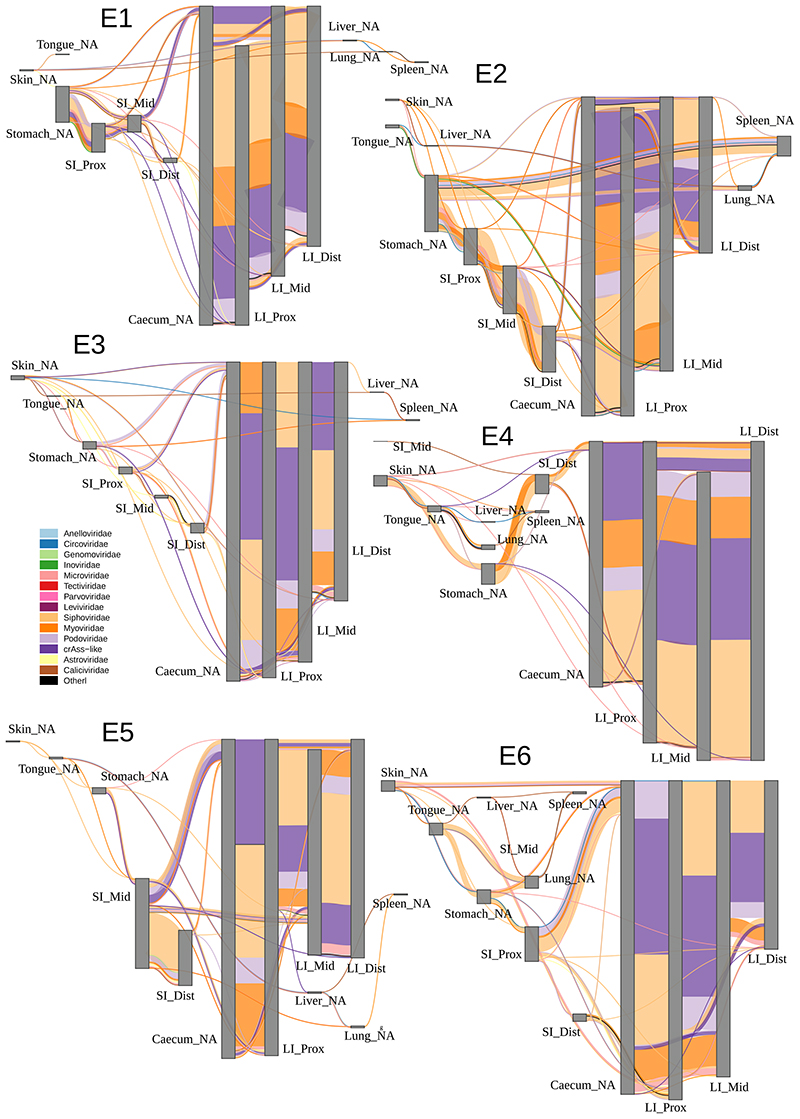
Sharing of viral genomic contigs between different anatomical sites in individual pigs (n = 6). Vertical grey rectangles height is proportional to viral richness (individual genomic contig counts) at each location, aggregated across luminal and mucosal samples; thickness of coloured connectors is proportional with the number of genomic contigs of each viral family shared between pairs of locations; SI, small intestine; LI, large intestine; Prox/Mid/Dist, proximal, medial and distal portions, respectively; unclassified genomic contigs were excluded; C, fraction of viral contig diversity from each organ represented in the distal LI.

**Extended Data Fig. 5:**
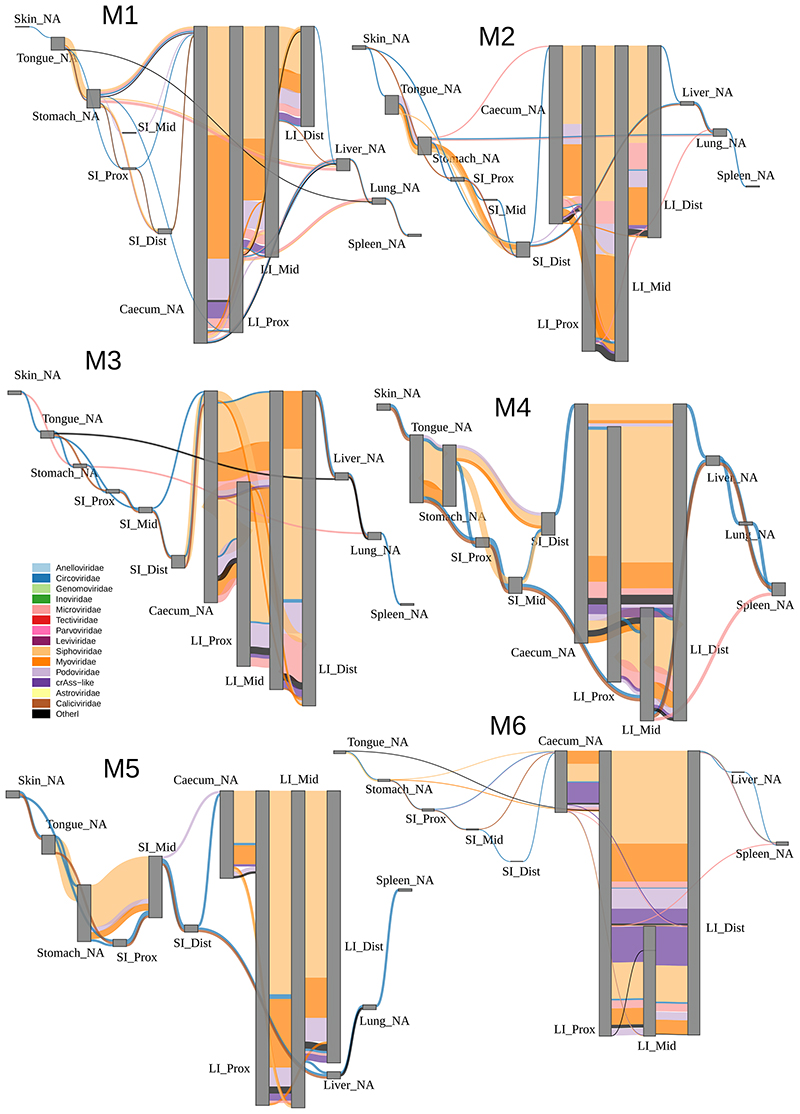
Sharing of viral genomic contigs between different anatomical sites in individual macaques (n = 6). Vertical grey rectangles height is proportional to viral richness (individual genomic contig counts) at each location, aggregated across luminal and mucosal samples; thickness of coloured connectors is proportional with the number of genomic contigs of each viral family shared between pairs of locations; SI, small intestine; LI, large intestine; Prox/Mid/Dist, proximal, medial and distal portions, respectively; unclassified genomic contigs were excluded; C, fraction of viral contig diversity from each organ represented in the distal LI.

Having observed only this partial separation of GIT sites by virome composition, we reasoned that there should be extensive sharing of individual viral species/strains between multiple GIT sites in each of the animals. To investigate this, we returned to the level of individual viral contigs and visualised their sharing between organs in a particular sequence. Agreeing with the individualised nature of gut viromes demonstrated above, patterns of viral contig sharing between different organs were also unique, not only between pigs and macaques, but also between individual animals within each cohort (Extended Data Figs. 4-5). Despite that, common trends in viral sharing between organs could also be easily observed.

**Extended Data Fig. 6:**
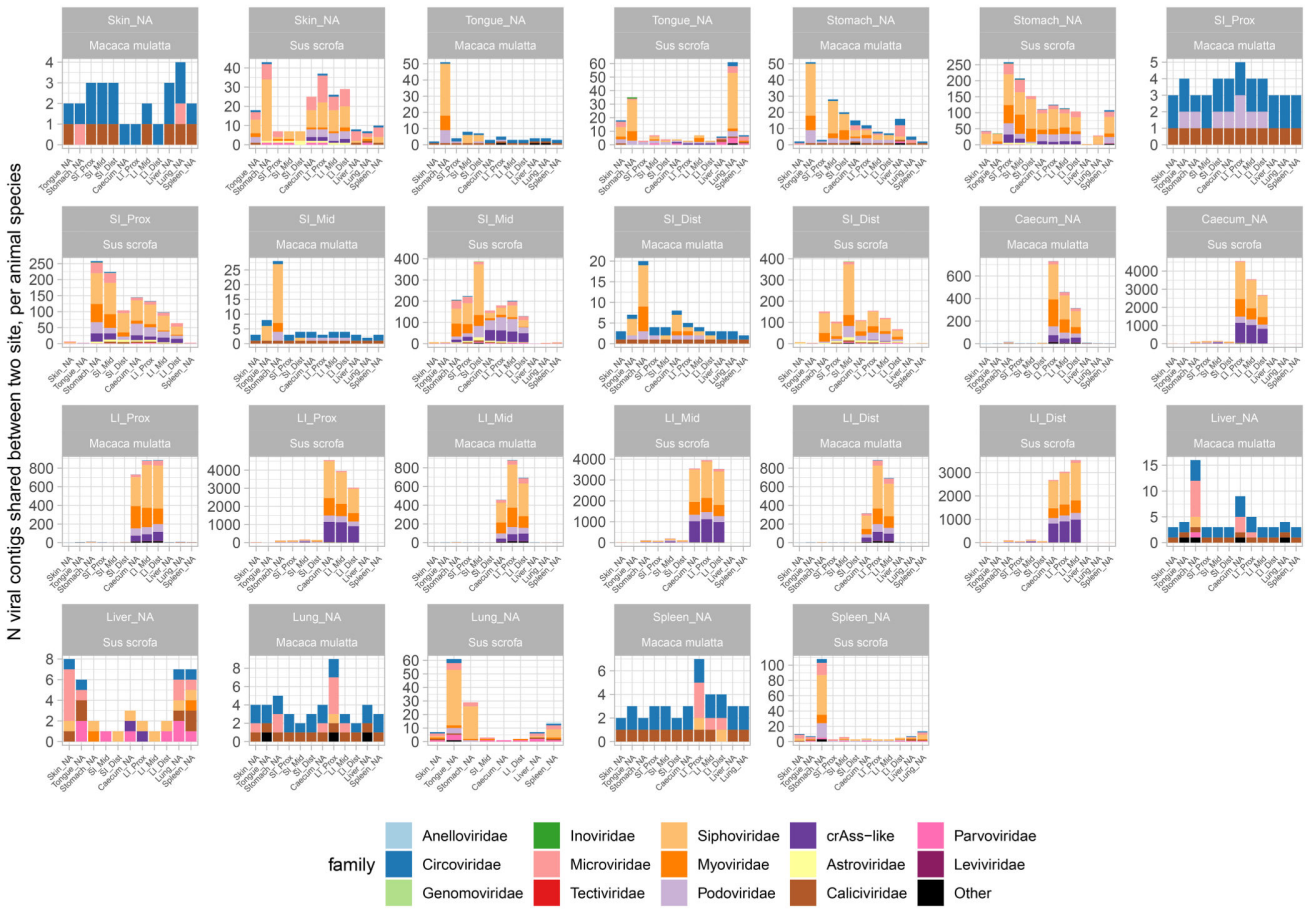
Numbers of viral genomic contigs shared between pairs of organs in pigs and macaques. Numbers of shared contigs are expressed as aggregate counts of unique contigs shared between sites across all animals for each of the two species; SI, small intestine; LI, large intestine; Prox/Mid/Dist, proximal, medial and distal portions, respectively.

**Fig. 3:**
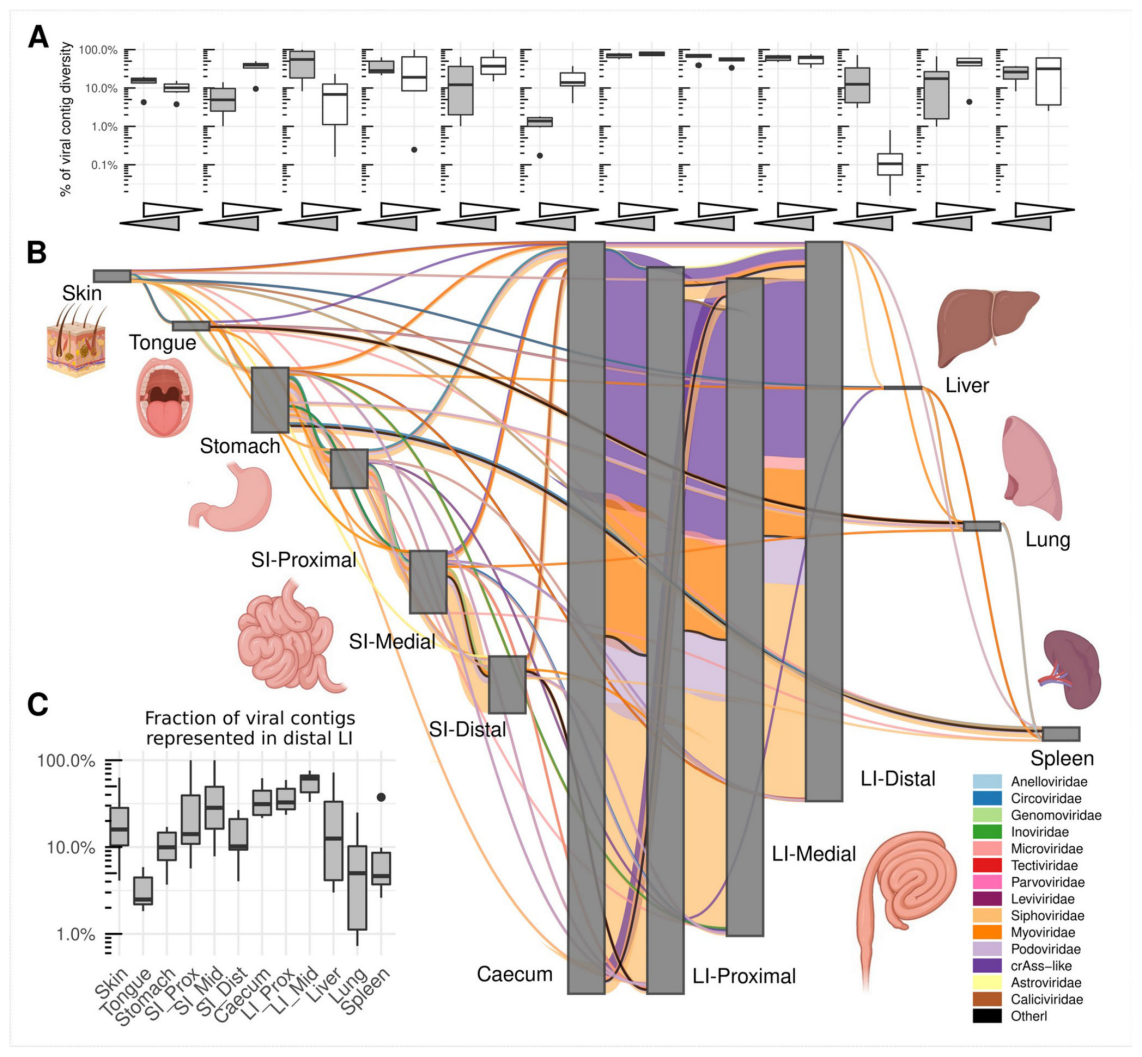
Sharing of viral genomic contigs between different anatomical sites in pigs (n=6). **A**, fraction of viral contig diversity shared between pairs of sites in both directions (white arrows/boxplots are forward direction, grey are reverse), in the order indicated in panel B; Boxplots are standard Tukey type with interquartile range (box), median (bar) and Q1 – 1.5 × IQR/Q3 + 1.5 × IQR (whiskers). **B**, aggregated map of viral contig sharing across six animals; vertical grey rectangles height is proportional to viral richness (individual genomic contig counts) at each location, aggregated across luminal and mucosal samples; thickness of coloured connectors is proportional with the number of genomic contigs of each viral family shared between pairs of locations; SI, small intestine; LI, large intestine; Prox/Mid/Dist, proximal, medial and distal portions, respectively; unclassified genomic contigs were excluded; **C**, fraction of viral contig diversity from each organ represented in the distal LI; Boxplots are standard Tukey type as above.

**Fig. 4:**
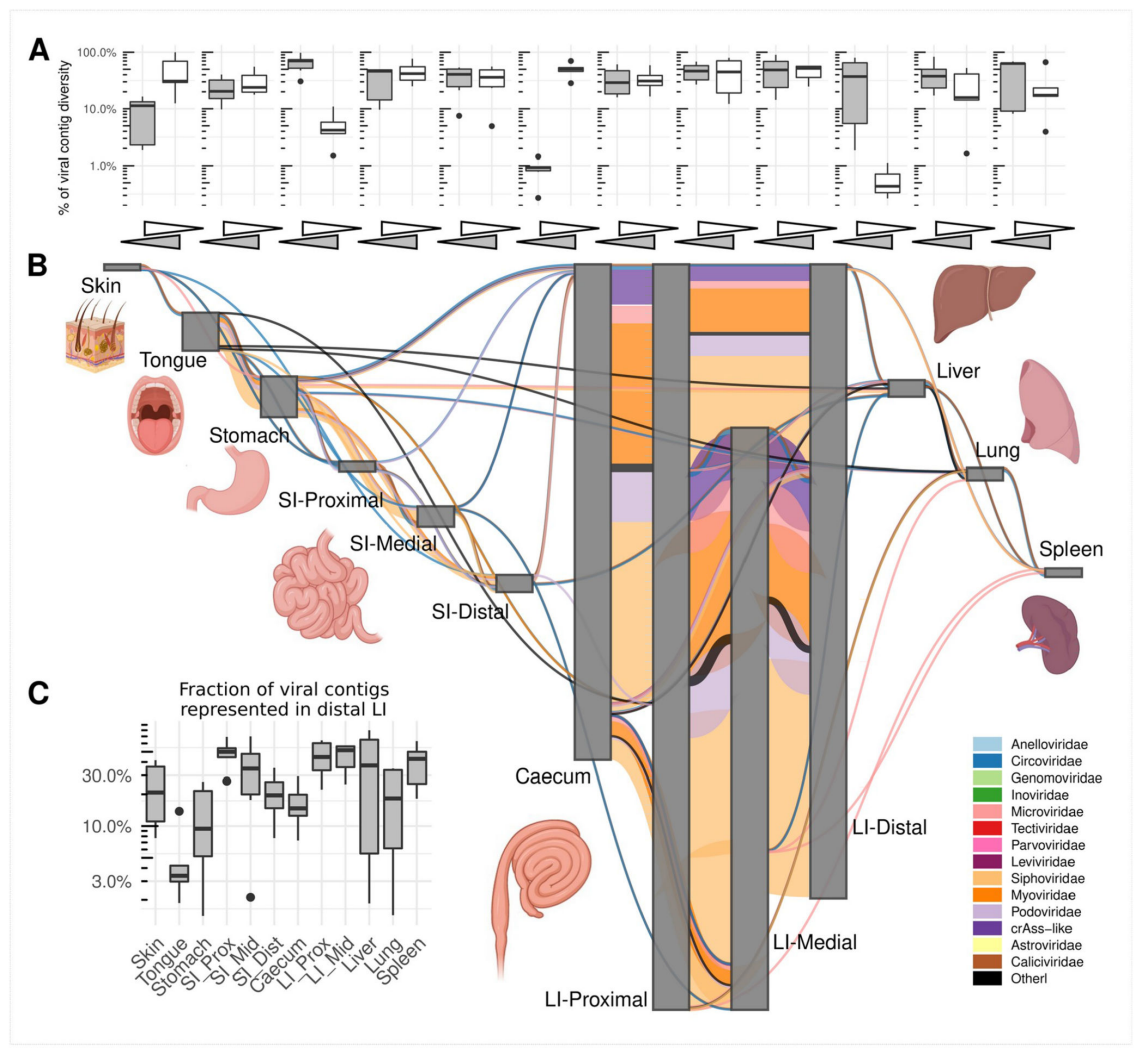
Sharing of viral genomic contigs between different anatomical sites in macaques (n=6). **A**, fraction of viral contig diversity shared between pairs of sites in both directions (white arrows/boxplots are forward direction, grey are reverse), in the order indicated in panel B; Boxplots are standard Tukey type with interquartile range (box), median (bar) and Q1 – 1.5 × IQR/Q3 + 1.5 × IQR (whiskers). **B**, aggregated map of viral contig sharing across six animals; vertical grey rectangles height is proportional to viral richness (individual genomic contig counts) at each location, aggregated across luminal and mucosal samples; thickness of coloured connectors is proportional with the number of genomic contigs of each viral family shared between pairs of locations; SI, small intestine; LI, large intestine; Prox/Mid/Dist, proximal, medial and distal portions, respectively; unclassified genomic contigs were excluded; **C**, fraction of viral contig diversity from each organ represented in the distal LI; Boxplots are standard Tukey type as above.

As shown in aggregate maps of viral contig sharing, summarizing the data from all pigs (Fig. 3) and all macaques (Fig. 4), high diversity populations of LI bacteriophages (Fig. 2B-C) are also efficiently shared between all locations in caecum and colon (Fig. 3 and Fig. 4). Summing up data from all pigs, for instance, >2,000 crAss-like phage and 65-130 *Microviridae* genomic contigs are shared between sites from the caecum to the distal colon (in luminal and mucosal samples together). Similarly, in macaques, 65-109 crAss-like contigs and 33-98 *Microviridae* were found shared between same anatomical sites. The same pattern was also true for tailed bacteriophage genomic contigs in the families *Siphoviridae*, *Myoviridae* and *Podoviridae* (Extended Data Fig. 6).

As a rule of thumb, >50% of viral contig diversity in pigs and >30% in macaques was shared between all locations in the LI. Extensive sharing of viral contigs was observed within SI of both animal species. By contrast, only 1-2% of pig distal SI viral diversity and <1% of the same in macaques was detectable in caecum samples (Fig. 3 and Fig. 4). This picture is however, complicated by the fact that some of the gastric and SI viral contigs that we failed to detect in the distal segment, were nevertheless found in caecum and/or in the lower segments of LI. This suggests that limitations in sequencing depth and/or strict criteria of contig detection might introduce some artificial gaps of contig detection across multiple anatomical sites in our data. Distal colonic samples (a proxy for faecal samples in our study), appeared to be good representatives of total viral diversity in the lower GI tract (>50% represented), and poorer representatives of the upper GI tract (~10% represented of gastric virome). Only a small fraction of tongue virome could be detected in the distal colon (Fig. 3 and Fig. 4).

**Extended Data Fig. 7:**
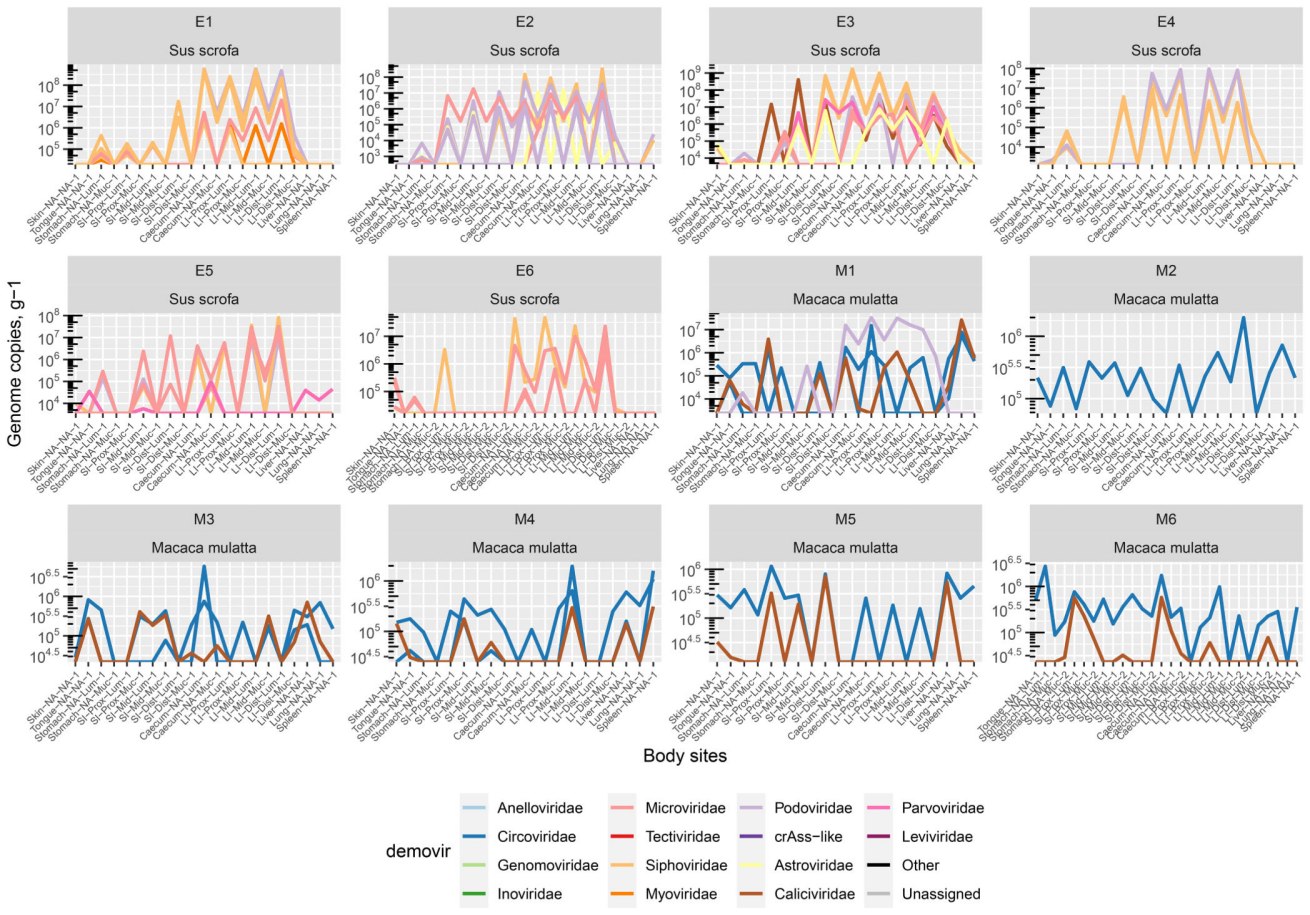
Absolute counts of some of the most ubiquitous viral genomic contigs present in pigs and macaques. Only contigs with >50% estimated completeness and shared between 6 or more sites in any of the animals are displayed. Each line corresponds to an individual genomic contig (potentially collapsing multiple viral strains). Colours are according to viral families. Each panel represent an individual animal.

Nevertheless, our data contains numerous examples of prokaryotic and eukaryotic viruses (genomic contigs with ≥50% estimated completeness) shared across six or more different anatomical sites. In pigs, such examples include *Astroviridae* and *Caliciviridae* species in luminal, *Parvoviridae* in mucosal samples and parenchymal organs, as well as numerous bacteriophage types across anatomical locations. In macaques *Circoviridae* and and *Caliciviridae* species were ubiquitously found (Extended Data Fig. 7).

Livers, lungs and spleens of both animal species, shared with the GIT sites not only the genomes of eukaryotic viruses (*Circo*-, *Calici*-, *Parvoviridae*) but also small genomes of *Microviridae* phages and other phage genomic contigs (Figs. 3-4; Extended Data Fig. 4-6). In the light of some recent publications, this can be interpreted as evidence for possible translocation of some digestive tract bacteriophages across healthy gut epithelia^61^, ending up in the internal organs (liver, lung, spleen), presumably via macropinocytosis, the portal vein (liver), lymphatic system, or perhaps via regurgitation of stomach contents (lung).

## Discussion

Recent studies have observed correlations in gut bacteriome and phageome composition and claimed associations between altered virome composition and GIT diseases in humans^31,32^. It has been speculated that phages could play a decisive role in controlling bacterial population density and structure via “kill-the winner” or similar types of ecological dynamics^62–64^. Indeed, in simplified microbiota models exponential growth of phage under optimal conditions can lead to the rapid collapse of sensitive bacterial populations^65^, resulting in cascades of knock-on effects in non-susceptible bacterial populations via inter-bacterial interactions^16^.

On the other hand, there is also convincing evidence that points toward a much less disruptive role of phages in microbiome composition, in that most numerically prevalent phage types are either temperate (existing in the form of prophages as well as free viral particles), or have evolved to support a long term, stable persistence in the microbiome with only limited effects on the density of bacterial host populations^66^. A number of potential persistence mechanisms have been proposed that includes phase variation of phage receptors in bacteria^20,28^ leading to herd immunity^21^, or physical segregation of mucus-embedded sensitive bacteria from luminal phages (“source-sink” model)^34^. It would be impossible to fully understand the dynamics of phage-host interaction and therefore the role of phages as either “drivers” or “passengers” in real-world complex microbiomes without having a detailed map of the virome in both temporal and spatial (biogeographic) dimensions. In this study we provide such a spatial map for two mammalian species, pigs and macaques.

From a technical perspective the study was designed to minimize the biases typically associated with virome analysis^42,47,67^. We used unamplified nucleic acids and assembly-based cataloguing of unclassified viruses, coupled with quantitation by comparison against a spike-in viral standard. We also clustered individual sequences into VCs to allow us to robustly detect and quantify both known and unclassified viruses with DNA or RNA genomes. Unlike in many previous studies^67^, we revealed an abundance of RNA viruses, including unclassified phages belonging to *Leviviricetes* class, and mammalian viruses belonging to the *Astroviridae* and *Caliciviridae* (Supplementary Information). Small ssDNA *Microviridae* phages were found to be a dominant group in the macaque colon, a finding that previously would have been dismissed as a DNA amplification bias^17,46^. A limitation of this assembly-based approach was, however, that we almost certainly missed some of the low abundance viruses seen in a previous study of the macaque virome^26^.

In the mammalian GIT, a number of factors may influence differences in the bacterial microbiota and virome between small and large intestines. Lower pH, higher oxygen tension, faster transit time and bile acid activity may limit bacterial growth in the SI, while a thicker mucus gel layer, slower transit time and shift to fermentation contribute to a large increase in microbial density in the LI^68^. As expected, the vast majority of phage biomass and diversity was concentrated in the colonic lumen, reflecting the dense community of bacterial hosts in that site. Upper GIT viromes were distinctly different and reflective of differences in bacteriome composition between different GIT regions (Supplementary Information). Direct correlations in density and composition between virome and bacteriome in the gut have been reported before^17^ and are consistent with the “piggyback-the-winner” ecological model^30^.

Interestingly, distal gut luminal viromes appeared to be very homogenous, from caecum to distal colon and compositionally much more reflective of an individual animal, than of a particular location in the colon. This confirms that inter-individual variability remains a hallmark feature of the intestinal virome^17^, even within these highly controlled environments. Recently, stochastic assembly effects have been shown to drive inter-individual variability in the bacterial microbiota in mice^69^ and this phenomenon should apply similarly to the virome in pigs and macaques.

Our results are in close agreement with research conducted on bacterial biogeography in the macaque gut, where Yasuda et al. observed predominantly inter-individual, and less location-specific, variation of luminal microbiota in jejunal, ileal, and colonic sites^70^. The same authors noted significant differences between luminal and mucosal microbiota in the same locations, with the latter being more influenced by biogeography than by an individual animal. In line with this, subsets of VCs appear to be specifically associated with both types of habitat (Supplementary Information). At the same time, mucosal samples show drastically reduced viral load and increased prevalence of viruses infecting mammalian cells.

These results may support a recently proposed “source-sink” model^34^ arguing that exclusion of bacteriophages from mucous layer creates a refuge for bacterial cells, allowing the co-existence of virulent phage and sensitive bacterial cells in close proximity. This apparently disagrees with an earlier “bacteriophage adherence to mucus” model (BAM)^37^, which argued that an accumulation of bacteriophages and an increased virus-to-microbe ratio (VMR ~ 39:1) in the mucus creates a barrier limiting bacterial invasion and segregating bacterial population to the luminal space. In the absence of quantitative data on bacteria, our study cannot testify to the VMR ratios in the lumen and mucosa. The BAM model therefore, can still accommodate our results, with a caveat that certain bacteriophages possessing Ig-like protein domains required for binding to mucus^37^ are equally abundant in the mucus and in the lumen, while phages lacking this ability are excluded into the luminal space. One can envisage complex scenarios of phage-host interaction in the GIT, with some phage-host pairs following “source-sink” dynamics, while others showing behaviours more conforming with the BAM model.

We observed extensive sharing of individual viral strains throughout the entire GIT. The most prominent examples were phages found continuously across multiple sites. For the majority of strains however, the continuous flow of phages from small to large intestine seems to be interrupted at the ileocaecal valve. This can be explained in part by drastic differences in composition (and presumably total biomass) of bacteriomes between SI and LI, which in turn support the growth of completely different phage populations. However, a complete extinction of small intestinal phages during passage from SI to LI seems unlikely, and therefore, the dilution effect, caused by vastly larger viral biomass supported by greater numbers of bacteria, combined with limitations imposed by sequencing depth, is a likely cause of the apparent disappearance of gastric and small intestinal phages in the caeca and LI.

Despite our original expectations, we could not definitively confirm a tendency for mucosal samples taken at 1cm distance to be closer in virome composition to each other than to other sites in the same anatomical region (Supplementary Information), which again suggests a relative homogeneity of virome along the proximal-distal axis within each region of the digestive tract. This observation calls for future longitudinal studies to examine viral flow and local temporal differences in virome composition in the gut.

Luminal samples from the distal colon, which can roughly be equated with faecal samples for the purpose of this study, are only representative of a fraction of the viral diversity present in different segments of the digestive tract. This is especially evident in the case of eukaryotic viruses, many of which are readily detectable in colonic mucosa (*Astroviridae* in pigs) or SI lumen (*Caliciviridae* in both pigs and macaques), and in parenchymal organs such as liver, lung and spleen, but not in the distal LI lumen. Interestingly, and agreeing with our earlier notion of virome individuality, each animal harboured a unique pattern of eukaryotic viruses, with regards to their taxonomic composition, strain variation and biogeographic distribution (Supplementary Information). The epidemiological and pathological significance of biogeographic distribution of these common viruses in porcine and murine GIT (in particular porcine *Astroviridae*^71^) is difficult to establish without further extensive population and longitudinal data collection.

Our findings in this study were largely consistent between pigs and macaques, despite differences in species, environment, diet and age. Notably, the pigs at three months were weaned and in early adolescence, while macaques were adults (5-12 years old). We note that all animals were female, thus preventing any determination of possible sex-effects on intestinal biogeography.

One of the interesting findings in this study was possible evidence of bacteriophage translocation from the gut into the systemic circulation and eventually parenchymal organs such as the liver, spleen and lungs. While animal dissection and sample collection for this study was conducted within a sanitary research environment, we could not achieve fully aseptic conditions in our pig facility. Therefore, it is possible some of the viral biomass in pig parenchymal organs that was orders of magnitude lower than was found in the gut could represent cross-contamination of solid organ samples. Nevertheless, we believe that this cannot fully explain our findings. Parenchymal organ viromes were dominated by eukaryotic viruses, and while phages present in these organs were specific strains shared with digestive tract viromes, they were not the most dominant strains. It has previously been demonstrated that at least specific phage types are able to adhere and translocate through the intestinal epithelial lining^37,61^. In our study, a tendency towards enrichment for smaller phages (family *Microviridae*) was observed in parenchymal organ viromes, which might indicate increased transepithelial diffusion of small viral particles. The exact fate of translocating phage and their systemic effects has so far remained unclear^72,73^, and our observations might be insightful for studying anti-phage immune responses^74^.

This work highlights that focussing on distal LI sampling (or faecal sampling) dramatically under-represents GI viral communities (particularly eukaryotic viruses), and points to consistent drop-out of upper GI viral communities in colonic samples. In addition to these findings, we detected some overlap between viral communities in parenchymal organs and the GIT which was not related to their overall abundance, suggesting that there may be some degree of specificity to viral translocation. Finally, we propose that this dataset and its accompanying methodology may provide an important catalogue of gut viruses and resource for future investigators in the field.

## Methods

### Ethical approval and study design

The study design was developed with consideration to the three Rs for ethical use of animals in science: replacement, reduction, and refinement. The proposed euthanasia only study was reviewed by the Animal Welfare Body (AWB) of University College Cork (Euthanasia Only Authorisation 17-005). With authorisation and under the remit of authorised and experienced personnel, the study was performed succinctly and with minimal distress to the animals involved. No statistical methods were used to pre-determine sample sizes but our sample sizes are similar to those reported in previous publications^26,70^. Data collection and analysis were not performed blind to the conditions of the experiments. No randomisation procedures were used and no data points were excluded from any of the analyses.

### Animal sampling procedures

#### (i) Sus scrofa domesticus – pigs

Six healthy female Landrace pigs (body mass approximately 30 kg, approximately 3 months of age) were sourced from a local farm in Cork, Ireland. All pigs were raised in a shared environment and on the same diet, although the relatedness of their parentage is unknown. Pigs were transported to the research facility on the morning they were to be euthanised, with two animals sampled back-to-back per day. Before euthanasia, work surfaces and necessary tools were disinfected using Virkon S disinfectant. Following anaesthetic overdose with Pentobarbital (150mg/kg) death was confirmed by an authorised person, and tissue samples were collected.

All biopsies (min. 3 cm × 3 cm) were minimally handled on site. Therefore, samples were not washed or stored in a buffer but placed directly into 50 ml Falcon tubes and stored on dry ice and then at -80°C. Initially, external biopsies of the tongue and skin were collected. Skin biopsies were taken from around the shoulder. Once external biopsies were obtained, pigs were rolled onto their back and a midline incision was performed from below the neckline of the animals to immediately preceding the genitalia. The complete gastrointestinal tract was removed from the abdominal cavity, with the connective tissue severed where required. Surgical thread was used to seal sections of the gastrointestinal tract. Two knots, approximately 2 cm apart, were tied tightly without severing the gastrointestinal tract. Subsequently, sections of the GI tract were separated by cutting between the tied knots that prevented the intestinal contents from leaking. Both the small and large intestines of animals were sealed in three approximately equal length sections to represent the proximal, medial, and distal regions. All sections of the GI tract were treated similarly. Briefly, an opening into the sealed GI tract tube was created and the contents removed before large representative sections of the bowel were cut and stored. Finally, stomach mucosa was from fundic region, and parenchymal organs were removed from the abdominal cavity of animals with large biopsies sections stored for later analysis. The processing time per animal was approximately 3 hours.

#### (ii) Macaca mulatta – rhesus macaques

Six healthy Indian-origin, female adult rhesus macaques aged 5-12 years with bodyweight 5.3 to 10.6 kg were used. All animals were born and raised in naturalistic multi-generational breeding groups at the Biomedical Primate Research Centre (BPRC), Rijswijk, The Netherlands, in comparable environments. All enclosures contained environmental enrichment and bedding to stimulate their natural behaviour. They were daily fed monkey chow pellets (Ssniff, Soest, Germany) in the morning, complemented with fruit and vegetables. Over a period of 5 months animals were euthanised using pentobarbital (70 mg/kg) following sedation with ketamin (10 mg/kg). The necropsy and collection of samples were done immediately after euthanasia.

For isolation and collection of macaque samples strict sterility protocol and safety procedures were used. The sterility of the necropsy table and the surgical instruments were assured using Virkon S, sterilization procedures and use of disposable scalpels. Macaque tissue samples were retrieved and stored similarly to the procedures outlined for pigs. For the collection of the parenchymal and intestinal samples disposable scalpels and autoclaved scissors and forceps were used. To avoid contamination, after opening the thoracic and abdominal cavity the first samples collected were from the parenchymal organs- liver, spleen, and lung following by the intestinal samples. After each animal, the table was thoroughly cleaned with hot water and detergent followed by disinfection by Virkon S, to prepare for the next animal. All samples were immediately placed on dry ice and stored at -80°C. Tissue samples were transported on dry ice to APC Microbiome Ireland for further processing

### Biopsy preparation procedure, VLP enrichment, and nucleic acid sequencing

GI and parenchymal organ sections of pigs and macaques were processed identically, in the same research facility, by the same team members, but on different days. Tissue samples were thawed on ice until completely defrosted. Excess faecal material on caecal and colon tissue sections were washed with sterile SM buffer (50 mM Tris-HCl; 100 mM NaCl; 8.5 mM MgSO_4_; pH 7.5). Tissue sections were stretched and pinned to a Styrofoam board using sterile syringes. Defined volume pinch biopsies of mucosal surfaces were collected with an endoscopic biopsy forceps. A “double-bite” of tissue samples at the same site ensured the accurate and complete loading of the forceps’ jaws. Mucosal pinches were removed from the forceps directly into pre-labelled Eppendorf tubes, filled with 400 μL of sterile SM buffer for processing.

To enable comparisons of viral load across biopsy samples, 10 μL of 10^7^ plaque forming units per millilitre of lactococcal phage Q33 were added to all samples. Additionally, Q33 in SM buffer or SM buffer-only were processed as negative controls. Fresh 0.5 M dithiothreitol (DTT) was prepared in 1 mL of SM buffer. A volume of 16 μL of the DTT stock was added to samples to achieve a final concentration of 20 mM, and samples were incubated at 37°C for 30 minutes. DTT was used to gentle solubilize mucin with minimal disruption of phage virions, as this disulphide bond reducing agent was previously demonstrated to release large quantities of non-mucin proteins from small intestine porcine preparations^75^. Host cellular debris and bacterial cells were pelleted by gentle centrifugation at 4000 g for 30 minutes at room temperature. Subsequently, 400 μL of liquid was aspirated and treated with 40 μL of DNase/RNase buffer (50 mM CaCl2; 10 mM MgCl2), 12 μL of DNase (manufacturer), 4 μL of RNase, and incubated at 37°C for 1 hour with intermittent inversion approx. every 15 minutes. Enzymes were inactivated by incubating at 65°C for 10 minutes.

Viral-enriched samples void of free nucleic acids were lysed using the QIAgen Blood and Tissue Kit following the manufacturers guidelines. However, samples were eluted in only 20 μL of AE elution buffer to increase the final concentration of nucleic acid obtained.

### Virome shotgun library preparation and sequencing

Reverse transcription (RT) reaction was performed using SuperScript IV First Strand Synthesis System (Invitrogen/ThermoFisher Scientific) with 11 μL of purified VLP nucleic acids sample and random hexamer oligonucleotides according to manufacturer’s protocol. Concentration of DNA purified using DNeasy Blood & Tissue kit (QIAGEN) was determined using the Qubit dsDNA HS kit and the Qubit 3 fluorometer (Invitrogen/ThermoFisher Scientific). DNA/cDNA yields varied between 0.05 and 29 ng/μl, with some samples being below detection limit.

Library preparation was carried out using Accel-NGS 1S Plus kit (Swift Biosciences) according to manufacturer’s instructions. Briefly, 20 μl of RT product (regardless of DNA concentration, as the kit is flexible with regards to the amount of input DNA) were taken for sonication after adjusting the volume to 52.5 μl with low-EDTA TE buffer. Shearing of unamplified DNA/cDNA mixture (variable amounts of DNA) was performed on M220 Focused-Ultrasonicator (Covaris) with the following settings: peak power of 50 W, duty factor of 20%, 200 cycles per burst, total duration of 35 s. All following steps were performed in accordance with the manufacturer’s protocol. A 0.8 DNA/AMPure beads v/v ratio was used across all purification steps in the Accel-NGS 1S Plus protocol. Post-preparation library QC (fragment length distribution and quantitation) was performed using Agilent Bioanalyzer 2100 with High Sensitivity DNA kit and Invitrogen Qubit. Dual-indexed pooled library was sequenced using 2×150 nt paired-end sequencing run on an Illumina NovaSeq platform at GENEWIZ (Leipzig, Germany).

In order to control for contamination of samples with exogenous viruses and viral nucleic acids, including lab reagent-derived and environmental, we also performed extraction from 400 μL of sterile SM buffer alone. Two samples were processed simultaneously with pig and macaque samples using the same protocol. Both of them yielded DNA/cDNA below detection limit after extraction, and only trace (insufficient for sequencing) amount of DNA was visible. To compensate for low yield, third sample was subjected to whole-genome multiple displacement DNA amplification (MDA, Illustra GenomiPhi V2 DNA Amplification Kit) as described before^17^.

### Analysis of virome shotgun sequencing data

Raw reads were processed using Cutadapt v2.4 to remove adaptor sequences. Trimmomatic v0.36^76^ was used for quality-based trimming and filtration of reads with the following parameters: ‘SLIDINGWINDOW:4:20 MINLEN:60 HEADCROP:10’. Reads aligning to mammalian genomes were identified using Kraken v1.1.1 in combination with *Macaca mulatta* (GCF_000772875.2_Mmul_8.0.1) and *Sus scrofa* (GCF_000003025.6_Sscrofa11.1) reference genome files.

Following removal of mammalian reads, levels of contamination with bacterial genomic reads were assessed using ViromeQC tool^77^. Reads were then assembled into contigs on a per sample basis using SPAdes assembler v3.13.0 in metagenomic mode with standard parameters^78^. Additionally, in attempt to assemble low-abundance genomes, reads were pooled by animal and assembled using MEGAHIT v1.2.1-beta^79^. All contigs > 1 kb were then pooled together and an all-vs-all BLASTn search was performed with *e-value* cut-off of ≤ 1E-20. Contig redundancy was removed by identifying pairs sharing 90% identity over 90% of the length (of the shorter contig in each pair) retaining the longest contig in each case.

To extract viral contigs from a background of bacterial contamination several selection criteria were used. Firstly, contigs aligning using BLASTn v2.10.0+^80^ against viral sequences in NCBI RefSeq database (release 208), Gut Virome Database^51^, JGI IMG/VR database (v3, release 12-10-2020)^50^, Gut Phageome Database^54^, and the recent human gut phage MGV database^55^, as well as our in-house database of crAss-like phage genomes (n=1,576), with at least 50% identity over 85% of contig length (*e*-*value* cut-off of individual hits ≤ 1E-10) were deemed as viral. Secondly, contigs that identified as viral using VirSorter2 pipeline^56^ with strict criteria (score ≥ 0.9 OR score ≥ 0.7 with at least 1 viral hallmark protein-coding gene present) were added. Completeness level of viral genomic contigs was determined using CheckV^81^ with default parameters. VirSorter2-identified viral contigs marked as prophages by CheckV (Provirus==Yes), were eliminated. Viral genomic contigs identified by these approaches constituted the final non-redundant viral sequence catalogue (n = 107,680).

Protein coding genes on viral contigs were predicted using Prodigal^82^ (-*meta* mode). Translated protein sequences were searched against PHROGs database^83^ of virus-specific protein family profile HMMs, using hmmscan (HMMER v3.1b2; *e-value* cut-off of ≤ 1E-5); viral protein sequences from NCBI nr (as of 02-11-2021) and viral RefSeq (release 208) databases and crAss-like phage proteins from an in-house database (n=7,356) using BLASTp v2.10.0+. (*e-value* cut-off of ≤1E-10). Circular genomic contigs were identified using LASTZ. G+C content was calculated using EMBOSS geecee.

Assignment of contigs to viral families was accomplished using Demovir script (https://github.com/feargalr/Demovir), as described before^17^. Clustering of viral genomic contigs (only for contigs with >3 kb in length) into viral clusters (VCs, approximately genus-level operational taxonomic groups) was done using vConTACT2 software^60^ with the following optional parameters: --*rel*-*mode Diamond* --*db* *ProkaryoticViralRefSeq85*-*Merged* --*pcs*-*mode MCL* --*vcs*-*mode ClusterONE*. Viral genomic contig catalogue was further manually curated to remove coliphage phiX174 genome (commonly used as a spike-in by sequencing facilities). Phage lifestyle (temperate vs. virulent) was predicted using BACPHLIP^84^ using 0.95 confidence threshold.

Remaining (non-viral) non-redundant contigs were assigned to bacterial taxa by performing BLASTn search against bacterial RefSeq (release 99) and HMP Reference Genomes databases. Taxonomic assignments were made at genus level, for contigs having 90% identity over ≥85% of combined alignment(s) length against a reference bacterial genome. CRISPR arrays were predicted on bacterial contigs and spacer sequences were extracted using PILER-CR v1.06^85^.

To predict the hosts of phage, data was aggregated from several sources. Firstly, previously predicted hosts for viral species included into IMG/VR database were assigned to viral contigs in our catalogue belonging to the same species (≥95% identity over ≥85% of viral genomic contig length, in accordance with MIUViG criteria for viral species demarcation in metagenomic sequence data^57^). Secondly, a search against an in-house CRISPR spacer database (derived from bacterial RefSeq [release 89] and HMP Reference Genomes) was performed as described before^17^ to assign hosts to viral contigs, missing close homologs in the IMG/VR database. In a similar fashion, matches were found with CRISPR spacers encoded by bacterial contigs (with taxonomy assigned as described above) in the present study dataset. Lastly, BLASTn similarity of viral contigs to closely related ≥90% identity over ≥85% of viral contig length) prophages in bacterial genomes (RefSeq database of bacterial genomes, release 99; HMP Reference genomes database^86^) was used to assign hosts where neither IMG/VR nor CRISPR approaches were successful. Lastly, tRNA gene hits against NCBI nt database (release 28-11-2020) and bacterial RefSeq database (release 99) were used to predict hosts for cases where all other methods failed. At the VC level, host was assigned using the majority vote rule, after aggregating host predictions from individual viral contigs – members of a particular VC.

Quantitative analysis of viral metagenomic data was performed essentially as described before^17^. Quality filtered reads were aligned to the curated viral contig database on a per sample basis using Bowtie2 v2.3.4.1 in the ‘end-to-end’ mode. A count table of contigs versus samples was subsequently generated using SAMTools v1.7. Sequence coverage was calculated per nucleotide position per contig per sample using SAMTools ‘mpileup’ command. Read counts for contigs in samples showing less than a minimum of 1x coverage of 75% of a contig length, were set to zero^17^.

Absolute viral counts were calculated for viral genomic contigs based on comparison of their relative abundance with that of the externally added standard (lactococcal phage Q33). Only viral contigs with estimated completeness of >50% were taken into account based on an assumption that additional genomic fragments, which together constitute the remaining <50% portion of the complete genome, will not be counted and therefore will not artificially inflate the calculated total viral loads.

### Bacterial 16S rRNA amplicon sequencing

During the biopsy preparation procedure, the porcine and macaque biopsy samples were reduced by DTT followed by centrifugation to reduce host tissue and bacterial cells and enrich the viral-like particles. However, the bacterial-containing pellet was used as the starting material for complementary 16S rRNA analysis of bacterial communities associated with the same biopsy samples analysed with respect viromes. The preparation and sequencing of 16S rRNA gene V3-V4 segment libraries followed the procedure outlined previously^43^.

### Analysis of bacterial 16S rRNA amplicon sequencing data

Bacterial 16S rRNA amplicon sequencing data was processed using a pipeline based on USEARCH v8.1 (64 bit). Forward and reverse reads of 16S rRNA V3-V4 segment were merged together allowing for an expected error rate of <0.5 per nucleotide position at overlap. Merged sequences were truncated to remove forward (first 17 nt) and reverse (last 21 nt) 16S rRNA primers. Reads were then de-replicated and singletons were removed, followed by clustering into OTUs at 97% sequence identity level. Chimeras were removed using -*uchime*_*ref* function with *rdp_gold* reference database. Individual reads were then assigned to OTUs generated above at 97% sequence identity cut-off and read count matrix was generated. Finally, taxonomic assignment of OTUs was performed using RDP Classifier v2.12.

### Statistical methods

All statistical analysis of sequencing data was carried out in R environment v4.1.0. Descriptive statistical visualisations were created using ggplot2 v3.3.3. Network visualisations were created using igraph v1.2.6. Heat maps were produced using gplots v3.1.1. Sankey diagrams were made using networkD3 v0.4. Permutational multivariate analysis of variance was performed using the adonis() function in Vegan with Bray-Curtis distances. Virome β-diversity was visualised through canonical analysis of principal coordinates with Bray-Curtis distances [capscale() function in Vegan v2.5-7 with default parameters]. Comparison of Bray-Curtis distances between viromes within organs was done using Wilcoxon test with Benjamini-Hochberg corrections. VCs differentially abundant between organs, tissues and animal species were identified using ANCOM-II^87,88^ with Benjamini-Hochberg correction, α=0.05, and *w*_0_ threshold set at 0.7. For between-organ tests, individual animal was used as random effect variable and models were adjusted for tissue type (lumen vs. mucosa) as covariate. This was followed by *post hoc* ANCOM-II tests for specific pairs of organs. For between-tissue tests (lumen vs. mucosa) and between-species tests (macaques vs. pigs), models were adjusted for individual animal or organ type as covariate, respectively. Correlations between fractional abundances of individual viral genomic contigs (or VCs) and bacterial 16S rRNA OTUs (or genera) were calculated using Spearman rank correlation method with Bonferroni correction for multiple tests.

## Extended Data

## Supplementary Material

Supplemental Materials

## Data Availability

All data needed to evaluate the conclusions in the paper are present in the paper, Supplementary Information file, and the additional dataset available at https://doi.org/10.6084/m9.figshare.15149247.v2. Raw sequencing data are available from NCBI databases under BioProject PRJNA753514.

## Code availability

Source data and custom R code used in this study are available at https://doi.org/10.6084/m9.figshare.15149247.v2. Further information and requests for data, code and resources should be directed to and will be fulfilled by Prof. Andrey Shkoporov (andrey.shkoporov@ucc.ie) and Prof. Colin Hill (c.hill@ucc.ie)..
