# Supplementary material for: Viral biogeography of the mammalian gut and parenchymal organs
Source: Nat Microbiol. Author manuscript; Available in PMC 2023 Jan 9. (PMC7614033; doi:10.1038/s41564-022-01178-w)
Supplement: Supplemental Materials [file EMS159074-supplement-Supplemental_Materials.pdf]

Viral biogeography of gastrointestinal tract and parenchymal organs in two representative species of mammals

Andrey N. Shkoporov*†[1,2], Stephen R. Stockdale*[1], Aonghus Lavelle[1], Ivanela Kondova[3], Cara Heuston[1], Aditya Upadrasta[1], Ekaterina V. Khokhlova[1], Imme van der Kamp[1], Boudewijn Ouwerling[3], Lorraine A. Draper[1], Jan A.M. Langermans[3,4], R Paul Ross[1,2], Colin Hill†[1,2]

[1] APC Microbiome Ireland, University College Cork, Cork, Ireland

[2] School of Microbiology, University College Cork, Cork, Ireland

[3] Biomedical Primate Research Centre, Rijswijk, The Netherlands

[4] Department of Population Health Sciences, Veterinary Faculty, Utrecht University, Utrecht, The Netherlands

* These authors contributed equally to the study

† Corresponding authors: andrey.shkoporov@ucc.ie, c.hill@ucc.ie

Supplemental materials

Supplemental Results

Overview of virome sequencing results

Illumina NovaSeq sequencing of DNA and cDNA prepared from VLP-enriched fractions yielded 1.8B reads, or 6.2±5.8M per sample (median±IQR; **Fig. S1**, **Table S1**) after trimming and quality-based filtration of raw data. Aligning reads against mammalian genomes (*Macaca mulatta*, *Sus scrofa domesticus*, *Homo sapiens*, *Mus musculus*) eliminated 3.4±7.3M reads per sample (1.3B or 70.6% in total), originating from host DNA/RNA contamination.

Our simple viral nucleic acids extraction and sequencing protocol was aimed at an accurate and relatively unbiased representation of viral sequences in the mammalian gut. At the same time, it appears to be prone to considerable amounts of contamination from non-viral sequences. **Fig. S1A** shows that luminal samples from the large intestine deliver largest fractions of non-mammalian Illumina reads that can be aligned to viral genomic contigs. At the same time, mucosal samples from both animal species, parenchymal organ samples (liver, lung, spleen), skin, tongue and luminal small intestinal samples from macaques largely consist of reads corresponding to mammalian genomes, and/or unaligned reads (potentially bacterial). This can be interpreted as extreme scarcity of viral DNA leading to relatively higher sequencing of co-purified host genomic DNA or bacterial DNA. Further supporting that, virome enrichment score (ViromeQC score) showed linear correlation with the total viral load in luminal samples (calculated from relative abundance of reads aligned to an internal spike-in standard phage Q33; **Fig. S1B**). After removal of mammalian reads, fraction of reads aligned to the viral contigs catalogue was negatively correlated to fraction of reads aligned to single-copy bacterial chromosomal markers (as calculated by ViromeQC; **Fig. S1C**)

Altogether, 0.78±3.0M reads per sample (or 550M reads in total) survived removal of mammalian sequences and 0.16±0.77M reads per sample (240M in total) could be aligned to the viral contig catalogue. After re-running ViromeQC pipeline only on reads aligned to viral contigs catalogue, a dramatic improvement of levels of bacterial contamination (estimated through relative abundance of bacterial single-copy chromosomal markers) was observed (**Fig. S1D**).

Catalogue of viral genomic contigs from pig and macaque GIT

Non-mammalian trimmed and filtered Illumina reads were assembled into contigs using a combined approach, both from individual biological samples (metaSPAdes assembler) and from per-animal read pools (MEGAHIT). The non-redundant set of contigs was further decontaminated of non-viral sequences using a combination of approaches (similarity to nucleotide sequences from virus databases and *de novo* virus genome identification by VirSorter2 pipeline[1]). The final catalogue includes 107,680 contigs (**Ext. Data Fig. 1**; **Additional Dataset**), ranging in size from 1,000 bp to 285,911 bp (*Prevotella* phage Lak-A1[2] genome fragment) and representing both complete circular (n=100), nearly complete high-quality genomes (n=1,305), as well as smaller genome fragments (**Ext. Data Fig. 1**).

When aligned against the genome databases of cultured and characterised viruses (viral portion of NCBI RefSeq[3]), as well as databases of complete and partial viral genomes extracted from metagenomic data (crAss-like phage genomes[4]; Gut Virome Database[5]; Gut Phageome Database[6]; MGV database of human gut viruses[7]; Joint Genome Institute IMG/VR viral database v3[8]), a total of 58,573 contigs had sequence identity of ≥50% with previously reported sequences, over ≥85% of their length. Of them, 1,129 aligned to NCBI RefSeq entries, 2,301 aligned to crAss-like phages, 24,538, 39,882 and 29,222 were similar to GVD, GPD and MGV entries respectively, while 35,512 were similar with IMG/VR v3 sequences. At the same time, when a recently proposed threshold (≥95% identity over ≥85% of a contig length[9]) for delineation of uncultured viral species is used, the majority of contigs aligned to database entries appear to be novel viral species. Approx. 71.2% of contigs had no close relatives in the representative databases.

As shown in **Ext. Data Fig. 1A and B** only a small fraction of contigs represent complete or nearly complete viral genomes (MIUViG “high-quality”; n = 1,305), whereas the majority of genomes are highly fragmented. Nevertheless, high-quality genomes appear to be among the most abundant in the virome samples and recruit highest proportion of Illumina reads (**Ext. Data Fig. 1C**). Of these high-quality contigs, 146 could be recognised by BACPHLIP as virulent bacteriophages and 181 as temperate.

Taxonomic assignment of viral genomic contigs to viral families revealed that, although the vast majority of them (n=93,526, **Ext. Data Fig. 2**) could not be reliably classified, the highest percentage of identifiable contigs belong to tailed bacteriophages (*Siphoviridae, Podoviridae, Myoviridae*[10,11], and crAss-like phages[12]; together 13,455 contigs) and small phages of the family *Microviridae* (n=473). Of interest is the presence of three unique *Leviviridae* genomic contigs. These ssRNA bacterial viruses were shown to be highly diverse and omnipresent, but are often overlooked in the metagenomic studies of gut viromes[13]. In addition to these highly diverse phage populations, both animal species carry a small core of eukaryotic viruses, *Astroviridae*,

Caliciviridae, *Circoviridae*, *Cruciviridae*, *Genomoviridae, Herpesviridae*, *Picornaviridae* and *Parvoviridae*, often represented by complete or nearly complete high-quality genomic contigs.

A reagent control sample (produced by MDA) contained a number of contigs (n = 87) overlapping with pig and macaques samples. Apart from a few larger contigs (uncultured crAss-like phage fragment, 26,469 bp; Lactococcus phage 28201 fragment, 18,975 bp; Lactococcus lactis prophage fragments, 15,121 and 5671bp), the rest of the contigs were small genomic fragments < 5kb. All contigs detected in the reagent control sample were subtracted from the entire dataset.

Viral diversity across individual animals and along the GIT longitudinal axis

Aligning Illumina reads back to the contigs catalogue resulted in recruitment rates which differed depending on the anatomical location and type of the sample (mucosa vs. luminal content). Samples taken from the upper GIT, parenchymal organs and mucosal tissue invariably produced low fractional counts of reads which could be aligned to the viral catalogue (**Fig. S1A**). This is largely explained by the differences in total viral loads (**Fig. 1**). Samples with higher viral loads (large intestine lumen) also tend to have larger fractions of reads aligned to the viral contig catalogue, despite higher α-diversity of viruses in those samples (**Fig. 2B-C**). Family-level composition of aligned viral reads is presented in **Ext. Data Fig. 2** and **Fig. S2** and is discussed in the main text. Differences in viral α-diversity between anatomical locations were dramatic, from one or a few viral genomic contigs dominating the virome of small intestine and parenchymal organs to many thousands of contigs in the large intestine lumen and mucosa samples (e.g. in pig E6 and macaque M6, **Fig. S3B**). Despite this high diversity in the lower gut, there was a tendency for a single dominant virus in many of the samples: such as a large genomic contig (234 kb) classified as complete *Myoviridae* phage with unknown host in caecal lumen and mucosa of macaque M6 (relative abundance of 0.36); or a 38.7 kb complete genomic contig of a *Siphoviridae* phage predicted to infect *Lactobacillus* in the large intestine of the same animal (**Fig. S3B**).

In order to find out whether the high diversity of viruses in the large intestine was a product of artificial inflation due to highly fragmented assemblies, we employed a recently published CheckV[14] tool to identify proportions of Illumina reads aligned to high-quality/complete genomic contigs on one hand, and small genome fragments on the other. As shown in **Fig. S4A,B** there was a tendency for large intestine samples to include even higher proportion of high-quality genomic contigs, compared to other body sites, therefore ruling out assembly fragmentation as a source of increased α-diversity in the lower gut.

As discussed in the main text, the extent of virus sharing between individual animals within each of the two species was quite low. Unlike the metagenomic analysis of bacteriomes, which is typically conducted at the level of OTUs, with taxonomic classifications being more robust at genus level[15], viral metagenomic is inherently strain-level, given the rapid evolution and diversity of viruses in the microbiomes[5,6,16]. It is known from human studies that strain-level diversity of the gut virome/phageome is very high and that only a small core of viral genomes is typically shared between unrelated individuals[17–20]. Strain level diversity of bacterial hosts, host genetics, individual dietary habits and co-habitation are factors, typically used to explain diversity and individual specificity of human viromes[19,21,22]. It was therefore surprising to see that relatively homogenous populations of animals, kept in the same facility and fed with a standardised diet, displayed high

level of individual virome variation. Only 23-35% viral contigs were present in more than one animal in pig and macaque cohorts, respectively, with 1.4-2.2% being shared by all members of a cohort. Comparisons of sparse, zero-inflated metagenomic count matrices coming from such divergent viromes (**Additional Dataset**) are unlikely to reveal any common biological signal[20]. To overcome this we used vConTACT2 algorithm[23] to cluster individual viral genomic contigs, both high-quality and fragmented into Viral Clusters (VC), provisional taxonomic units identified based off gene sharing patterns. VCs identified by vConTACT2 roughly correspond to the level of genus in current ICTV (International Committee on Taxonomy of Viruses) taxonomy[9,10]. From 3,770 VCs obtained from clustering of 12,262 individual viral contigs (singletons were not taken into account), 50-72% were shared between at least two animals out of six in a cohort, respectively, and 2.7-9.0% were shared across all animals of the same species (**Fig. S4C-D**). Out of 3,770 VCs identified in this study, 968 appear to be shared across the two animal species.

As outlined in the main results section, permutational analysis of variance using ADONIS revealed that within-species inter-individual virome variation was as strong (9.6% variance explained in Adonis with Bray-Curtis distances, $p = 0.001$) as the variation between different organs within a particular animal species (9.4% variance explained, $p = 0.001$), and higher than the fraction of variance explained by animal species (4.2%, $p = 0.001$). The interaction of between-organ differences and between-animal differences could explain 30% of variance in the data ($p = 0.001$ in Adonis). By contrast, differences between luminal and mucosal viromes, with or without interaction with individual animal or animal species, only accounted for 1.0-1.9% ($p = 0.001$) variance in the dataset, depending on the model tested. We then proceeded to identify most significant covariates, associated with virome variance using constrained analysis of principal coordinates with Bray-Curtis distances. Together with organ-specific variation (largely driven by the separation of caecum and LI-specific virome), total viral load and α-diversity metrics (e.g. Shannon diversity) explain a sizeable amount of total virome variance ($R^2 = 17.2\%$, $p = 0.001$ in a permutational ANOVA of a CAPSCALE model). In the CAPSCALE ordination, both the Shannon diversity and the total viral load were strongly associated with the first constrained axis that provides the greatest separation between organs (44% variance explained). This confirms that certain compositional differences between the stomach, small intestine, large intestine, and other organs occur along the ascending gradient of virome diversity and total viral load (**Fig. 2A**).

Viral diversity at neighbouring mucosal sites versus distant sites in the GIT

In a single pig (E6) and macaque (M6) we performed additional paired sampling of mucosal lining (1 cm apart) in order to reveal the level of local mucosal virome variance, and to determine whether viromes of two closely located mucosal sites are significantly more alike compared to more distantly spaced sites within the same alimentary tract organ. Bray-Curtis distances, based on virome composition at the level of individual viral genomic contigs, were calculated for all possible within-animal combinations of two anatomical sites, and compared between paired mucosal samples taken from proximal, medial and distal segments of SI and LI, caeca and stomach on one hand (pig E6 and macaque M6), and mucosal and luminal samples taken from different segments of the SI and LI on the other hand (**Fig. S3A**).

For paired mucosal samples there was a tendency for caecal and LI sites to be more compositionally conserved than SI and stomach, and more related to each other than mucosal samples taken from different segments in LI. These differences, however, do not reach statistical significance due to the small number of samples analysed. At the same time, between-segment differences in LI mucosa were shown to be considerably greater than between-segment differences in LI luminal virome ($p = 0.001$ in Wilcoxon test with Benjamini-Hochberg correction), but less pronounced than between-segment variation of the SI mucosal virome ($p = 0.043$ in Wilcoxon test with Benjamini-Hochberg correction). This findings are in line with a previous report on macaque gut bacteriome[24] which observed greater biogeographic variation of mucosal sites, compared to luminal contents.

These results can at least partly be explained by much lower levels of viral load and α-diversity in the SI sites compared to the LI, and potentially higher level of stochasticity of qualitative and quantitative virome composition revealed by the metagenomic sequencing, due to a low DNA input (**Fig. 1; Fig. 2B; Fig. S3B**).

While paired mucosal samples may not show more significant similarity between them than that with more distant mucosal sites, hierarchical clustering of all samples by β-diversity reveals that many individual pairs were in fact closer to each other than to any other sample from the same animal. This effect was especially evident in LI and caecum (but not SI) mucosa of macaque M6 (**Fig. S3C**), and SI and caecum (but not LI) mucosa of pig E6 (**Fig. S3D**).

Differentially abundant viral contigs

In order to identify the VCs driving separation between different GIT organs, as well as between luminal and mucosal viromes, the following tests was applied. Since organ-specific differences and inter-individual variability of virome were identified above as the strongest factors contributing to overall virome variance, we used mixed effect model implemented in ANCOM-II package[25,26], specifying organs as a fixed effect and individual animal as a random effect variable (while also adjusting for an effect of tissue – lumen vs. mucosa). In this test, 217 VCs were differentially abundant between organs across the two animal species (ANCOM $\alpha=0.05$ with Benjamini-Hochberg correction; ANCOM significance threshold $w_0 = 0.7$; **Fig. S5**). We then proceeded with a series of *post hoc* ANCOM-II tests to identify VCs that were discriminatory between pairs of organs in the following order: Skin-Tongue ($n = 8$), Tongue-Stomach ($n = 12$), Stomach-SI ($n = 6$), SI-Caecum ($n = 52$), SI-LI ($n = 117$).

In a similar manner, we tested a model using either the tissue type (lumen vs mucosa) or animal species (macaques vs pigs) factor as a fixed effect, adjusting for either inter-individual differences between animals or between-organ differences. We identified 20 VCs discriminatory between luminal and mucosal sites across all GIT organs, and 97 VCs differentiating the two species of animals (**Fig. S6-7**). Importantly, 11 VCs were significantly overrepresented in the mucosal samples, compared to matched luminal samples, highlighting potential existence of a mucosa-specific virome.

We then tried to put the differences in virome composition between the alimentary tract organs into the context of similar differences displayed by the bacteriome[24]. To do that, we looked

for rank correlations of fractional abundance between differentially abundant VCs (n = 217), which approximately correspond to the taxonomic level of genus, and bacterial genera (n = 349; **Fig. S8**). We set a threshold at the level of strong to very strong relationships (Spearman $\rho \geq 0.6$, $p < 0.05$ with Benjamini-Hochberg correction). By doing this we were able to observe 275 correlated VC-bacterial genus pairs in domestic pigs and 89 in rhesus macaques. With very few exceptions (2 and 10 negatively correlated pairs in pigs and macaques, respectively), all detected correlations were positive. As shown in **Fig. S9**, bacterial genera, involved in positive correlations with organ-discriminatory VCs, often represent taxonomic groups that are hallmarks of the microbiome of a particular segment or organ in the alimentary tract. For example, in macaques bacterial genera such as *Treponema, Desulfovibrio,* Ruminococcus, *Blautia, Dorea, Faecalibacterium, Gemmiger,* and *Oscillibacter*, themselves typical for the LI bacteriomes, were involved in dense networks of positive correlation with VCs, strongly associated with the LI and caecal viromes. Bacterial genera such as *Veilonella*, *Leptotrichia*, *Fusobacterium* were linked with the upper GIT organs. Similar, but distinct, patterns of organ-specific viral and bacterial communities can be seen in pigs (**Fig. S9**). These findings further confirm the tight association of bacterial viruses with their bacterial hosts in the gut as suggested in previous studies[17,27,28].

Eukaryotic viruses shared between different anatomic locations in pigs and macaques

In both species of mammals, parenchymal organs, skin and the alimentary tract organs were found to share collections of eukaryotic viruses, belonging to at least five different viral families (*Astroviridae, Parvoviridae, Circoviridae, Caliciviridae, Anelloviridae*) both within individual animals, and across animals. While all five families make up the eukaryotic virome in pigs, macaque viromes are mainly composed of *Circoviridae* and *Caliciviridae* (**Fig. S10**).

For the purposes of this study, clusters of closely related individual viral genomic contigs were collapsed together when reaching the threshold of 90% nucleotide identity over 90% of a shorter contig length. Therefore, true diversity of viruses at strains, and perhaps even species level cannot not be revealed using this approach. Nevertheless, up to 32 non-redundant viral genomic contigs (up to 13 per viral family; **Fig. S11**) could be identified in a single animal. These included contigs with high level of nucleotide similarity (≥95% identity over ≥85% of viral genomic contig length) to known viruses (Porcine parvovirus 5, Porcine bocavirus 5/JS677, Adeno-associated virus 2), as well as genomic sequences of potentially novel viral species, falling below that threshold of similarity to existing species.

Patterns of viral colonisation in all animals were individual, with respect to both the composition of eukaryotic viruses detected and their distribution between organs and total eukaryotic virus loads. Some viral species seem to be conserved across animals (e.g. 7,507 nt *Caliciviridae* genomic contig – Sapovirus NongKhai-24/Thailand – present in all macaques and pigs), whereas others are individual-specific (e.g. a 3,074 bp *Circoviridae* genomic contig found only in macaque M1). The majority of eukaryotic viral load in pigs was concentrated in SI, with viral counts exceeding 10^8 genome copies g^{-1} in some cases. By contrast, in macaques eukaryotic viruses seem to be mainly associated with LI, as well as SI and parenchymal organs (**Fig. S11**).

The majority of detected viral species tend to be broadly distributed in each of the individual animals, rather than be concentrated to a particular body site. Extreme examples of such ubiquitous

viruses include a 2,224 bp genomic contig of *Circoviridae* found in all macaques, and almost equally abundant in GIT sites from tongue to distal LI, as well as in the spleen, lung, liver and on the skin (**Fig. S10**, **Fig. S11**).

Virus-to-bacteria correlations in the GIT

Having obtained evidence for the continuous presence of individual viral genomic contigs along the proximal-distal axis of GIT, we attempted to detect correlations in fractional abundances of individual viral contigs and bacterial OTUs across all anatomical locations on a per animal basis. Overall, we detected 45±49.5 (median±IQR) viral contigs per animal (540 unique contigs in total) strongly associated with bacterial OTUs (Spearman $\rho \geq 0.6$; $p < 0.01$ with Bonferroni correction). Detected correlations were overwhelmingly positive (**Fig. S12-S13**).

To further confirm these observations and filter out cases of correlation unrelated to direct phage-host relationships, we performed a more focussed analysis of the phage-host pairs for which correlation with a particular host agrees with host prediction (to genus level) from viral sequence analysis (20 viral contigs and 58 bacterial OTUs). Such prediction was based on: a) host assignment for closely related viral homologs from IMG/VR database[8]; b) CRISPR spacer matches[6]; or c) detection of homologous prophages in members of the same bacterial genus (**Additional Dataset**). With this focussed subset of viral contigs, the observed correlations with bacterial OTUs were always positive. Next, we examined whether lysogeny (integration of phages into host DNA) could explain strong correlations between some phages and their predicted bacterial hosts. To do that we determined the proportions of phages predicted to be temperate, virulent, or without confident lifestyle prediction (based on BACPHLIP tool[29]), among high-quality viral genomic contigs correlated to their predicted hosts, uncorrelated, and having no host prediction. The ratios of temperate/unknown/virulent phages (~8.0/77.5/14.5%) between these groups appeared to be varied insignificantly ($p = 0.21$ in χ^2 test).

This observation agrees with earlier reports of high temporal stability of the gut virome, with a correlation of the viral composition with the composition of the bacteriome, and supports the idea that the replication of the majority of phages in the gut environment proceeds at levels, or via mechanisms, that do not lead to collapse in numbers of the corresponding host bacteria[17,30]. Cases of out-of-sync fluctuations of fractional abundance in phage-host pairs, implying the growth of a bacterial population at sites where phage activity was low, and collapse sites where the corresponding phage was actively replicating, were rare (**Fig. S12-S13**). These findings are indicative of low phage predation control of bacterial population densities in the gut, and agree with the “piggyback-the-winner” ecological model[31], which postulates that high bacterial densities in microbial ecosystems, such as the mammalian GIT, favour temperate or temperate-like behaviour of the resident bacteriophages, as opposed to stricter population control and “kill-the-winner” dynamics imposed by phages in low density marine environments[32].

Supplemental Figures and Tables

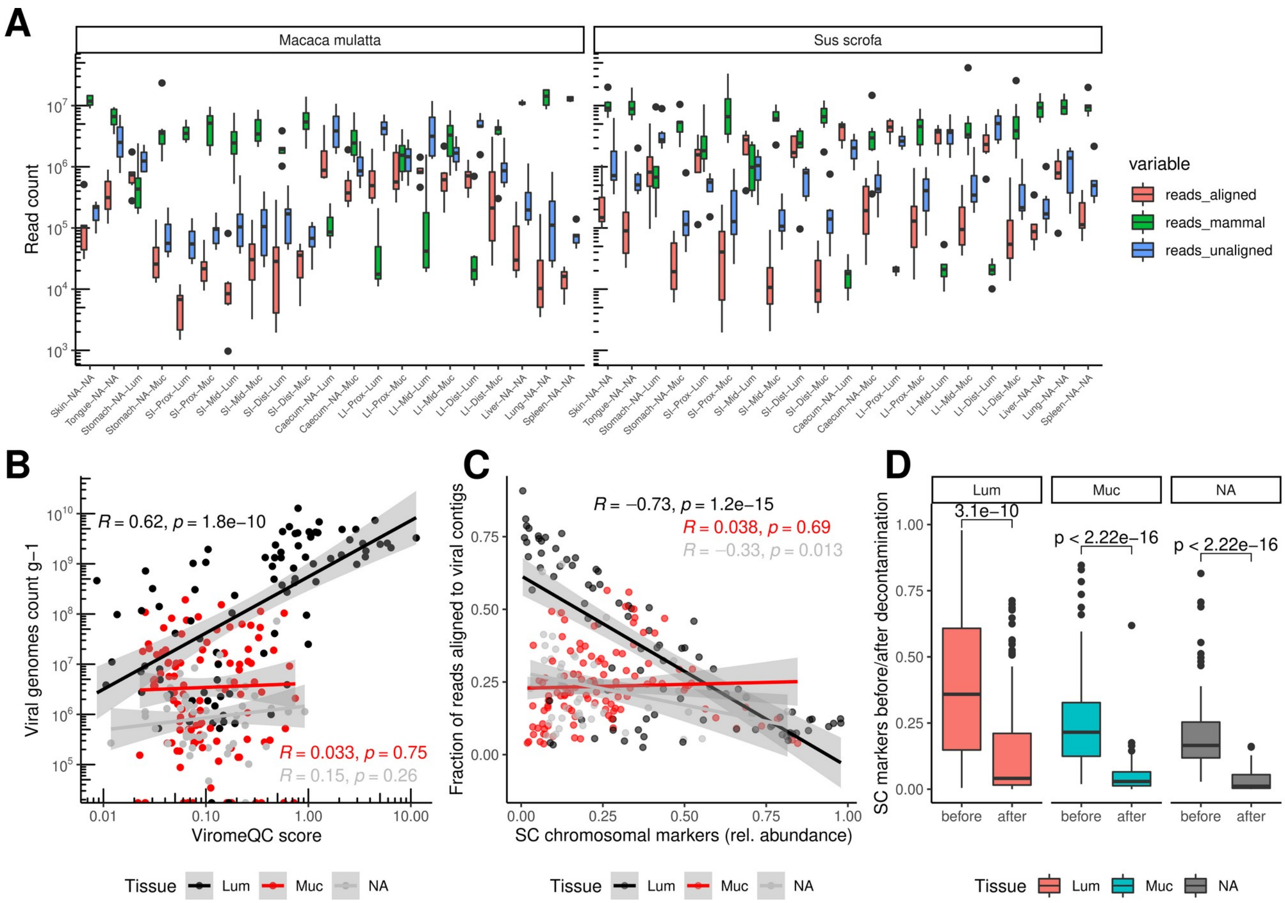

Fig. S1. Total read counts and non-viral sequence contamination. A, Illumina read counts per anatomical location per animal host species (n = 6 in each) after separating of reads aligned to mammalian genomes ("reads_mammal": *Macaca mulatta*, *Sus scrofa domesticus*, *Homo sapiens*, *Mus musculus*), and reads aligned to viral contig catalogue ("reads_aligned"), from "reads_unaligned" – putative bacterial reads; **B**, positive correlation (Pearson's r, two-tailed test) of ViromeQC score (degree of virome enrichment) with calculated viral genome counts per g in luminal gut samples (before removal of reads aligned to mammalian genomes); regression lines are given with 95% confidence intervals (grey shading); **C**, negative correlation (Pearson's r, two-tailed test) of fraction of reads aligned to viral contigs with relative abundance of bacterial DNA (single-copy genomic markers) in luminal samples (after removal of reads aligned to mammalian genomes); regression lines are given with 95% confidence intervals (grey shading); **D**, reduction in levels of bacterial contamination (single-copy bacterial genomic markers) in Illumina reads (GIT luminal samples n = 90; GIT mucosal samples n = 111; other tissue samples n = 59) after selecting them by alignment to the viral contigs catalogue (Wilcoxon signed rank test, two-tailed). Boxplots in panels A and D are standard Tukey type with interquartile range (box), median (bar) and Q1 – 1.5 × IQR/Q3 + 1.5 × IQR (whiskers).

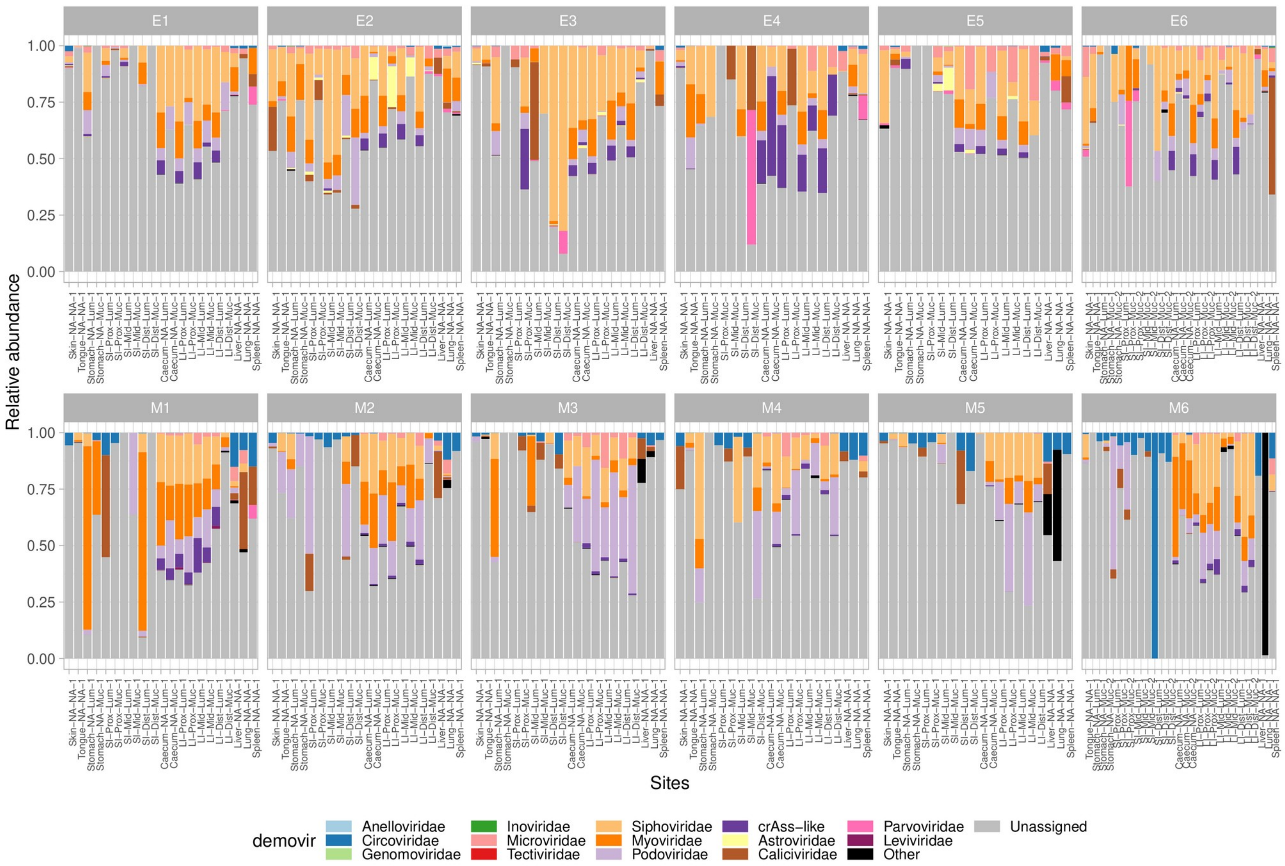

Fig. S2. Fractional abundance of viral contigs grouped at the viral family level. Fractional abundance is shown as a fraction of reads aligned to every contig out of the total number of reads aligned to the viral contig database. "Other" category includes families *Adenoviridae* (2 contigs), *Cruciviridae* (2 contigs), *Herpesviridae* (7 contigs), *Picobirnaviridae* (3 contigs), *Picornaviridae* (2 contigs), and *Virgaviridae* (1 contig). Samples are grouped by individual animals (M1-6 for macaques, E1-6 for pigs).

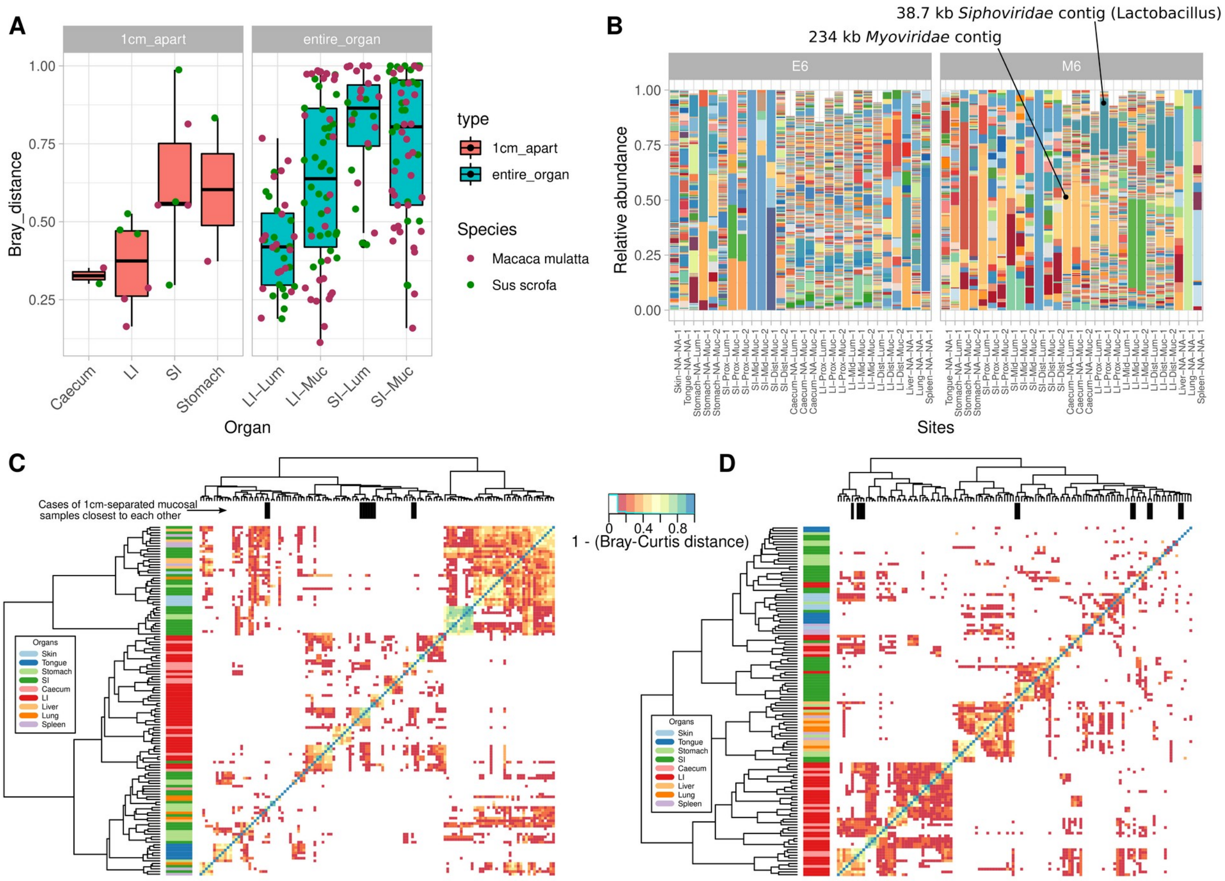

Fig. S3. Viral diversity at neighbouring mucosal sites versus distant sites in the GIT. A, Bray-Curtis distances between mucosal sites separated by 1 cm distance (“1cm_apart”, macaque M6 and pig E6) versus same distances between all combinations of mucosal and luminal sites (proximal, medial, distal locations) within an entire organ in each of the 12 animals separately; LI, large intestine; SI, small intestine; Lum, lumen; Muc, mucosa; differences did not reach statistical significance in two-tailed Wilcoxon tests with Benjamini-Hochberg correction; Boxplots are standard Tukey type with interquartile range (box), median (bar) and Q1 – 1.5 × IQR/Q3 + 1.5 × IQR (whiskers). **B**, Fractional abundance of viral contigs in macaque M6 and pig E6; fractional abundance is shown as a fraction of reads aligned to every contigs out of the total number of reads aligned to the viral contig database; only contigs with fractional abundance of > 0.01% in any of the samples are shown; colours are randomly assigned to each individual contig; **C** and **D**, pairwise Bray-Curtis distances between all mucosal and luminal sites in all macaques (C) and pigs (D); paired mucosal sites are highlighted with black bars on top; the left-hand side annotation bar represent different organs; trees representshierarchical clustering of sites based on reciprocal distance patterns.

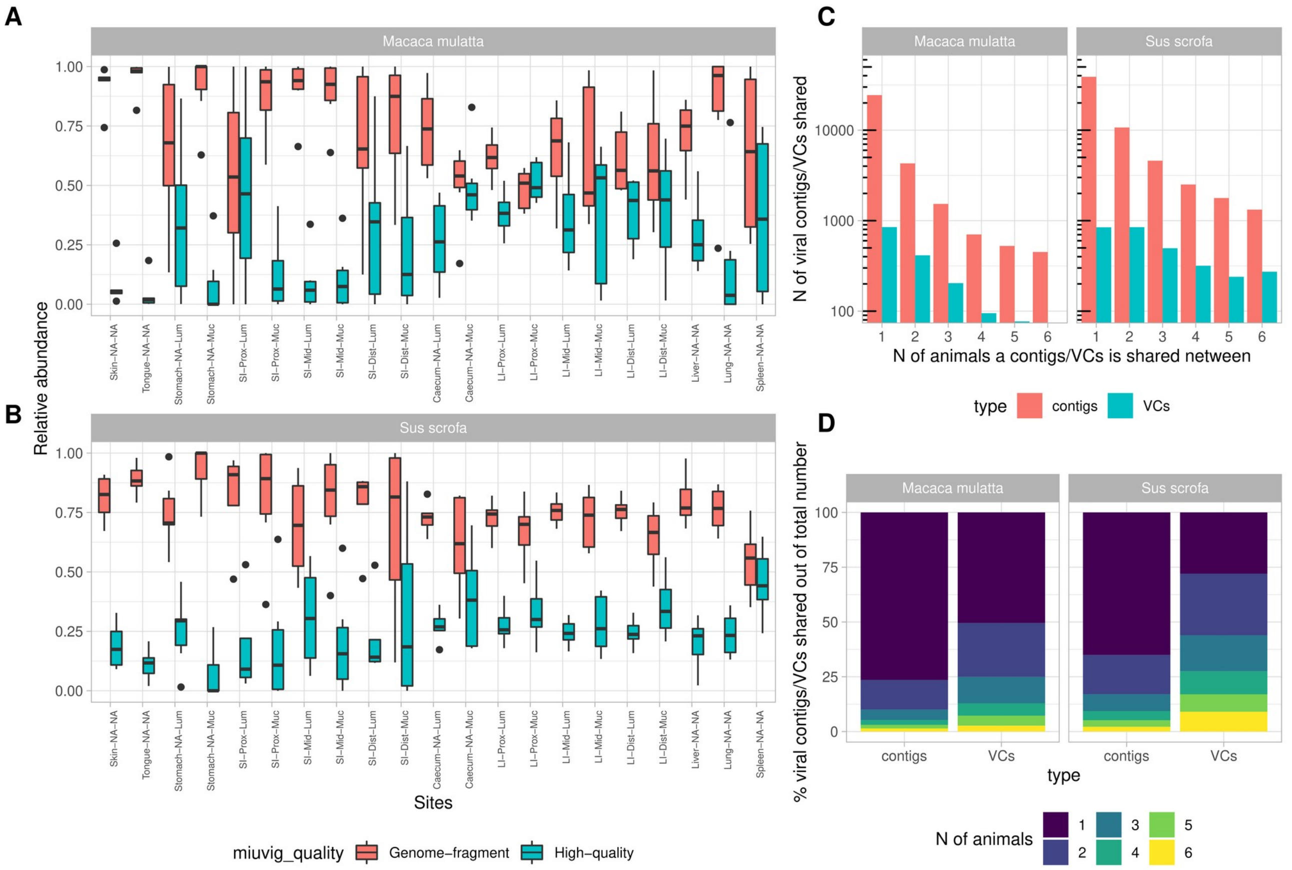

Fig. S4. Fractions of reads aligned to viral contigs of different quality levels, and sharing of viral contigs and clusters between animals. A and B, fraction of reads (out of total number of reads aligned to the viral genomic contig database) aligned to either high-quality contigs or smaller genome fragments (assigned by CheckV[14]) in different anatomical locations in pigs (n = 6) and macaques (n = 6); boxplots are standard Tukey type with interquartile range (box), median (bar) and Q1 – 1.5 × IQR/Q3 + 1.5 × IQR (whiskers). C, number of viral genomic contigs or viral clusters (VC) shared between individual animals in macaque and pig cohorts; D, percentage of viral contigs (out of 107,680) and VCs (out of 3,770) shared between certain number of animals within macaque and pig cohorts.

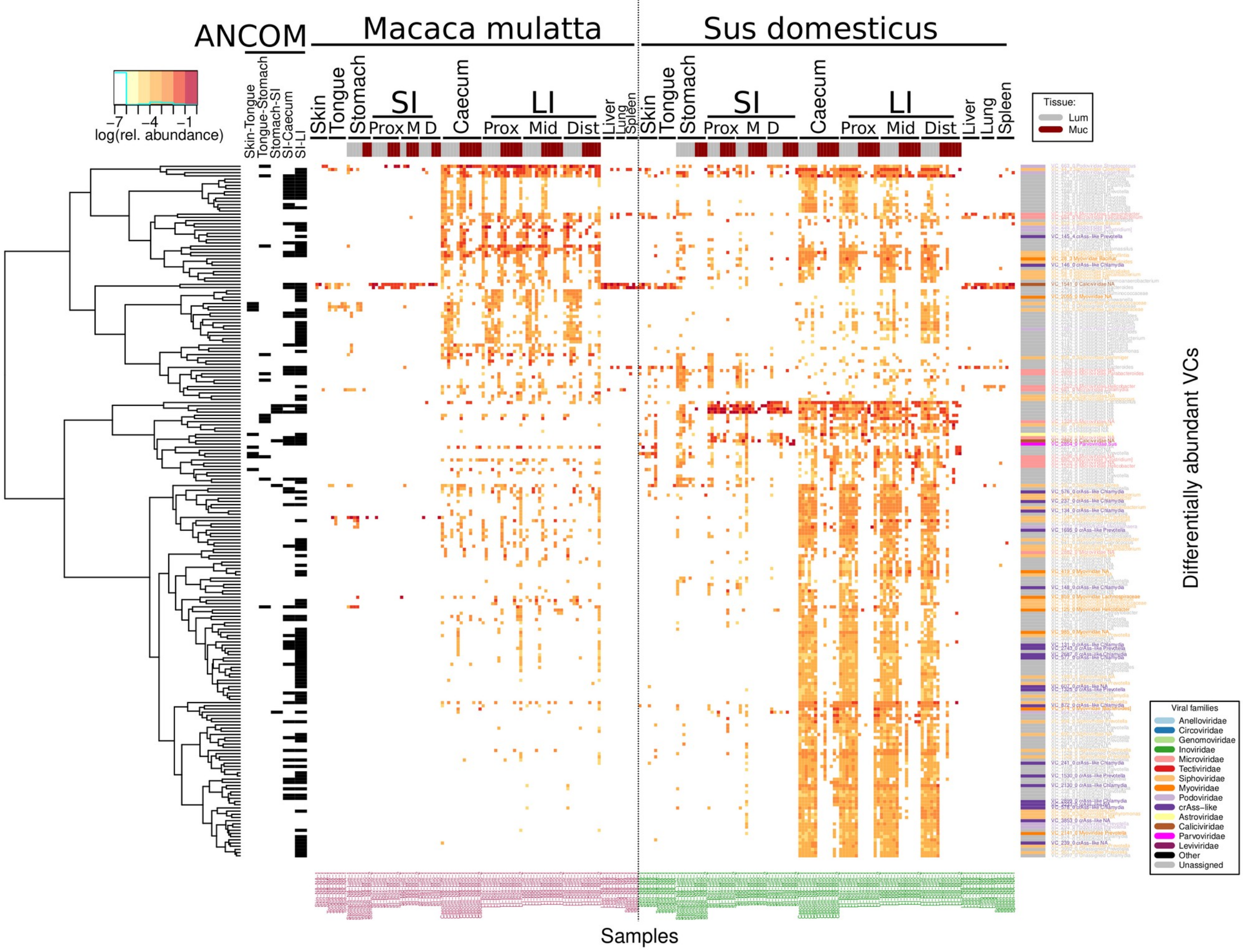

Fig. S5. VCs (n = 217) differentially abundant between GIT organs across two animal species. Differentially abundant VCs were selected using ANCOM-II test (α=0.05 after Benjamini-Hochberg correction; ANCOM significance threshold w_0 = 0.7). A series of post-hoc tests identified VCs (annotated with black bricks) discriminatory between the following anatomic locations: Skin-Tongue, Tongue-Stomach, Stomach-SI, SI-Caecum, and SI-LI. Columns are individual samples. Rows are VCs. Row labels contain VC name, classification to viral family level, predicted host (or NA, where not available). The top and the right-hand side annotation bars represent tissue types (lumen vs mucosa) and viral families of VCs respectively. the viral relative abundance data is plotted as log10-transformed values. Tree represents hierarchical clustering of VCs based on relative abundance patterns.

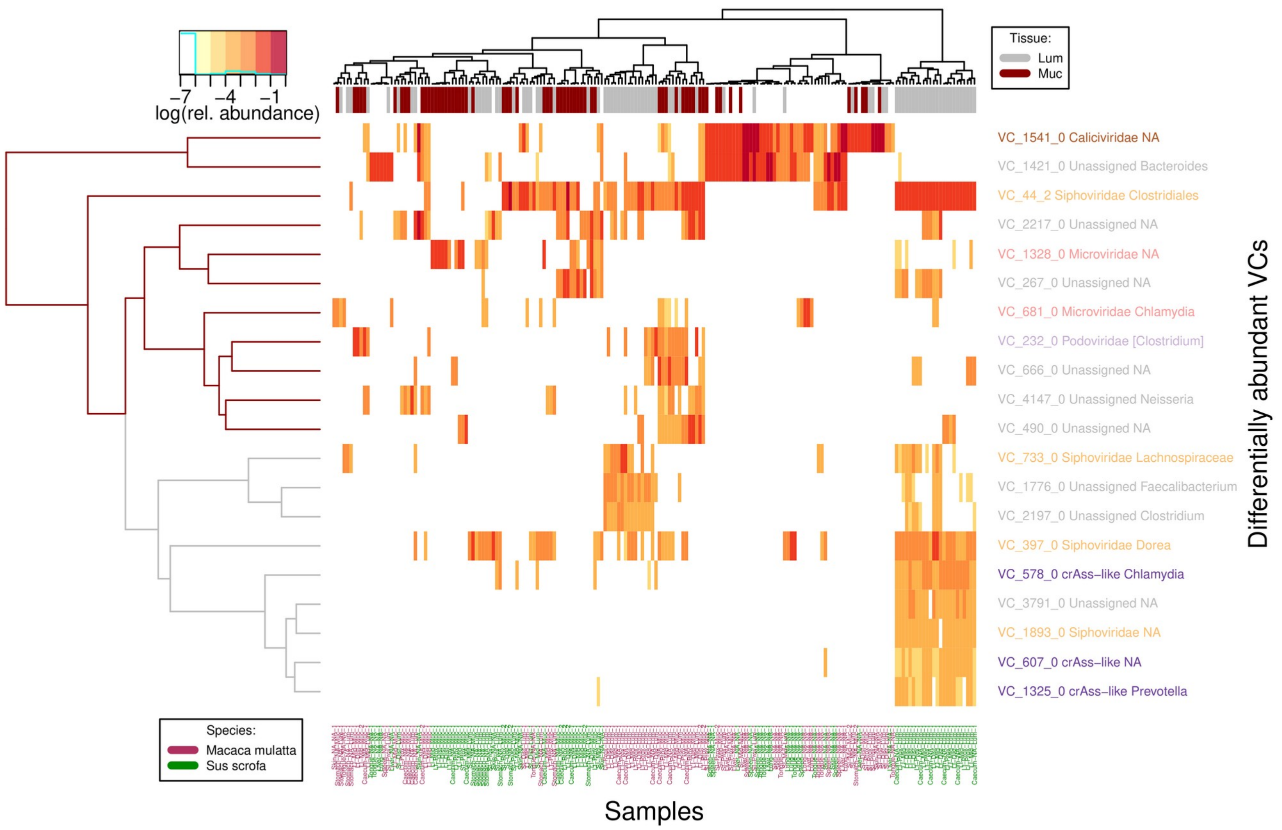

Fig. S6. VCs (n = 20) differentially abundant between mucosal and luminal sites in GIT organs across two animal species. Differentially abundant VCs were selected using ANCOM-II test, adjusting for inter-individual differences between animals (α=0.05 after Benjamini-Hochberg correction; ANCOM significance threshold w_0 = 0.7); the viral relative abundance data is plotted as log10-transformed values. Columns are individual samples. Rows are VCs. Row labels contain VC name, classification to viral family level, predicted host (or NA, where not available). The top and the right-hand side annotation bars represent tissue types (lumen vs mucosa) and viral families of VCs respectively. the viral relative abundance data is plotted as log10-transformed values. Tree represents hierarchical clustering of VCs based on relative abundance patterns (dark red branches are VCs enriched in mucosal samples, grey branches – luminal samples).

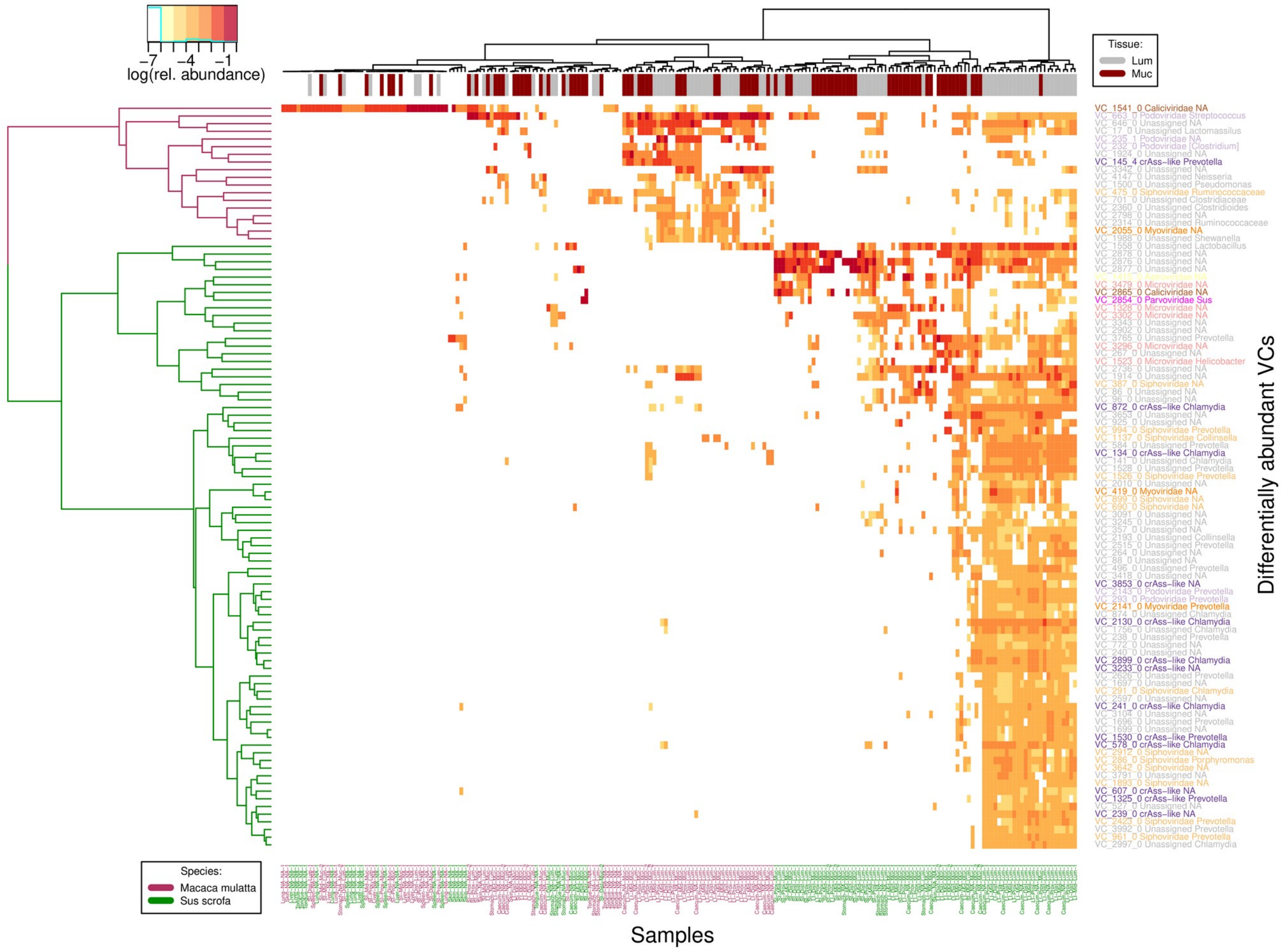

Fig. S7. VCs (n = 97) differentially abundant between the two animal species (pigs and macaques). Differentially abundant VCs were selected using ANCOM-II test, adjusting for between-organ differences (α=0.05 after Benjamini-Hochberg correction; ANCOM significance threshold w_0 = 0.7); the viral relative abundance data is plotted as log10-transformed values. Columns are individual samples. Rows are VCs. Row labels contain VC name, classification to viral family level, predicted host (or NA, where not available). The top and the right-hand side annotation bars represent tissue types (lumen vs mucosa) and viral families of VCs respectively. the viral relative abundance data is plotted as log10-transformed values. Tree represents hierarchical clustering of VCs based on relative abundance patterns (purple branches are VCs enriched in macaques, green branches – pigs).

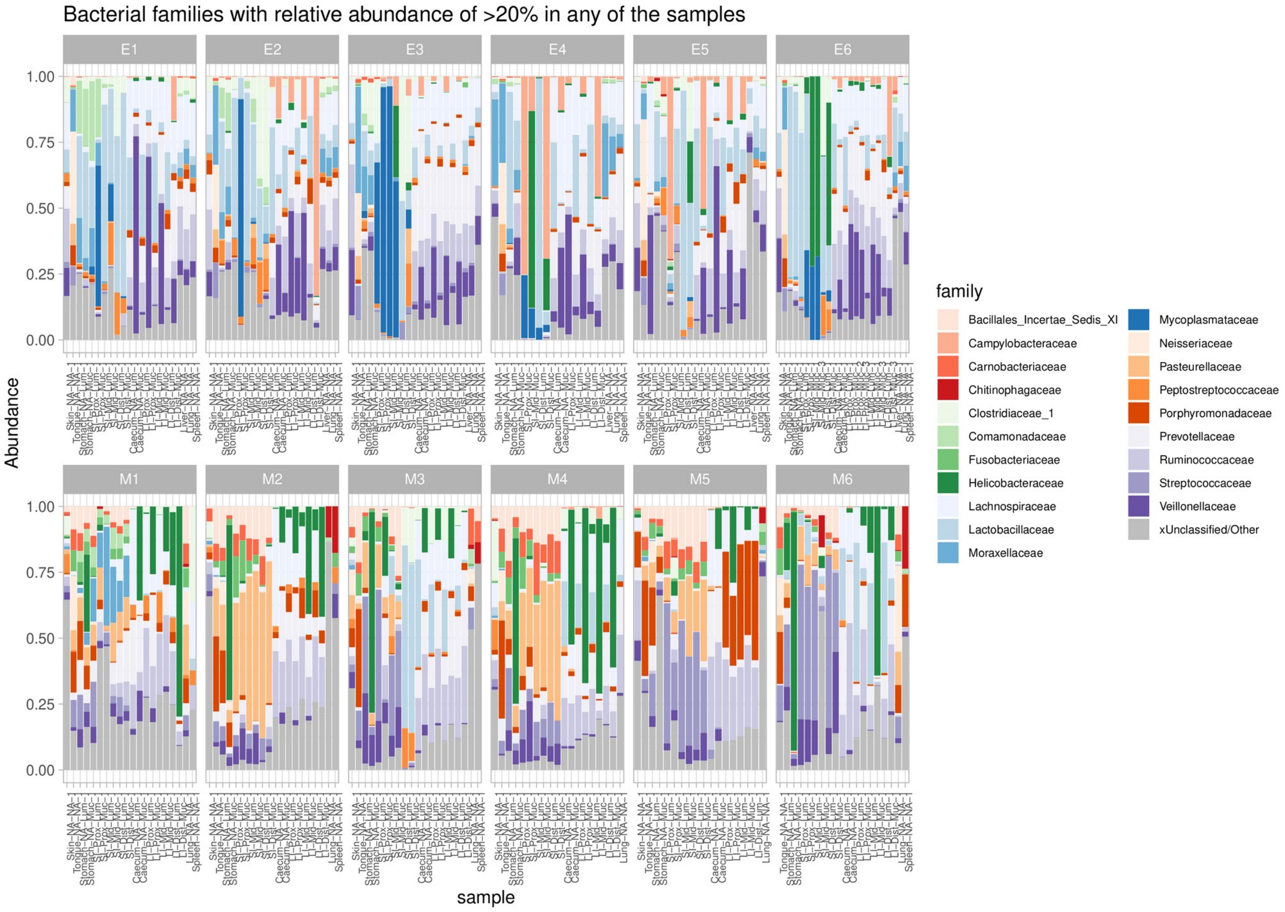

Fig. S8. Fractional abundance of key members of bacterial microbiota at family level (with >20% abundance in any of the samples) determined by 16S rRNA gene amplicon sequencing. Samples are grouped by individual animals (M1-6 for macaques, E1-6 for pigs). Colours indicate different bacterial families.

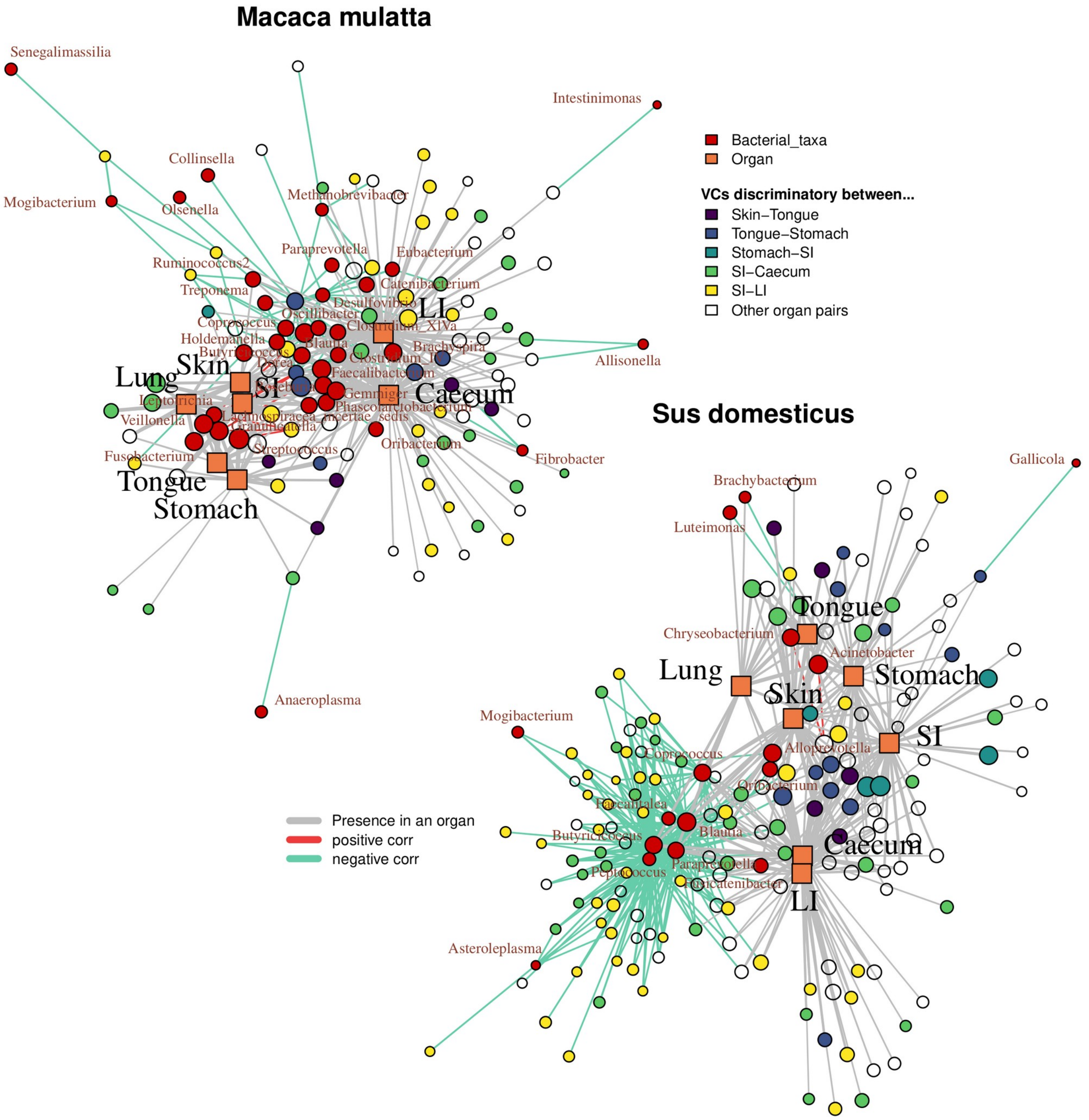

Fig. S9. Co-abundance networks based on Spearman rank correlation between fractional abundances of organ-discriminatory VCs and bacterial genera. In macaques, 89 strongly correlated pairs of VCs and bacterial genera were observed ($p < 0.05$ in two-tailed tests; $|\text{Spearman } \rho| \geq 0.6$). Similarly, in pigs 279 correlated VC-bacterial genus pairs were found. Network plots were built using iGraph and laid out using Fruchterman-Reingold algorithm; vertices represent organs (orange squares), bacterial taxa (red circles), and VCs discriminatory between different organ pairs; grey edges thickness is proportional to log10 fractional abundance in a given organ (only cases with relative abundance of ≥ 0.1% are shown); green and red edges represent positive and negative correlations, respectively.

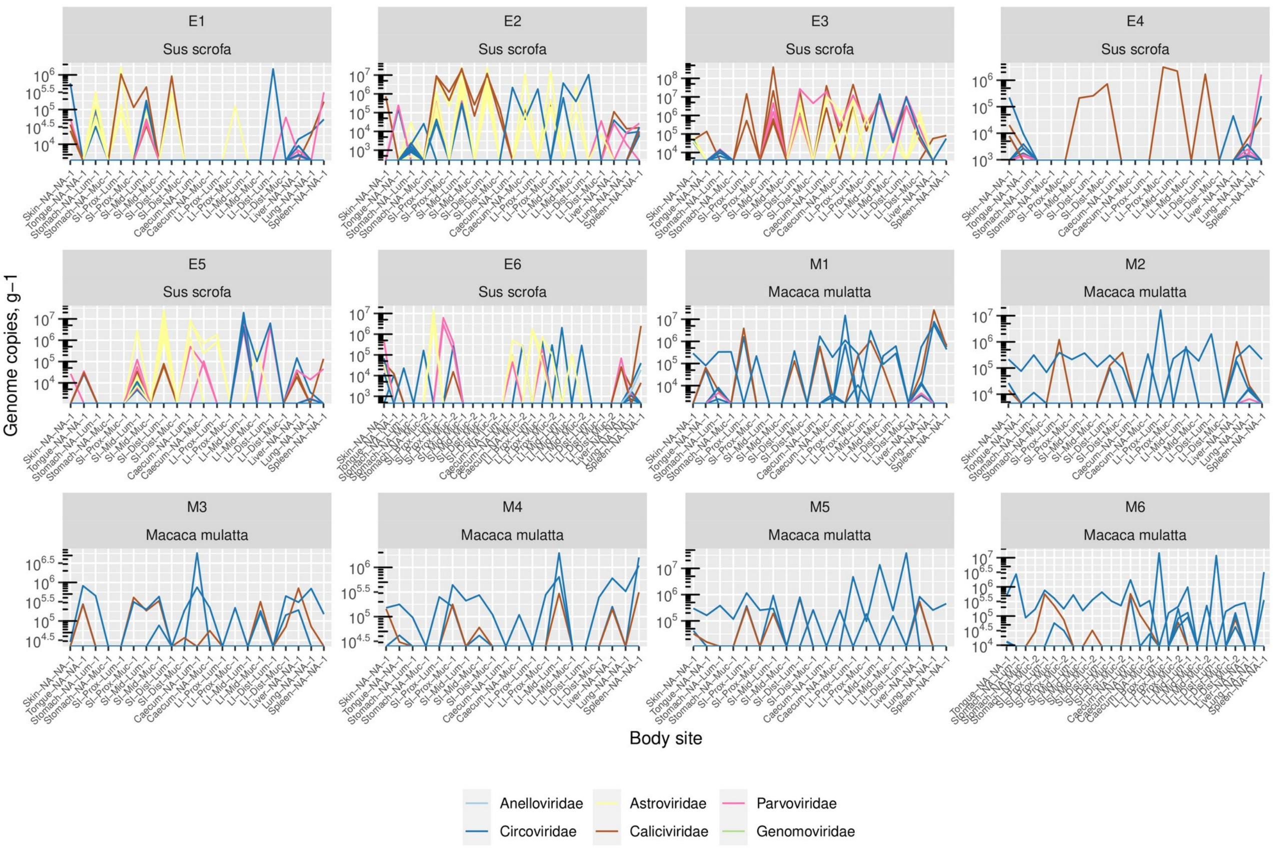

Fig. S10. Absolute counts of eukaryotic viral genomic contigs in all tested body sites in pigs and macaques. Only contigs with >50% estimated completeness are shown. Each line corresponds to an individual genomic contig (potentially collapsing multiple viral strains). Colours are according to viral families. Each panel represent an individual animal (M1-6 for macaques, E1-6 for pigs).

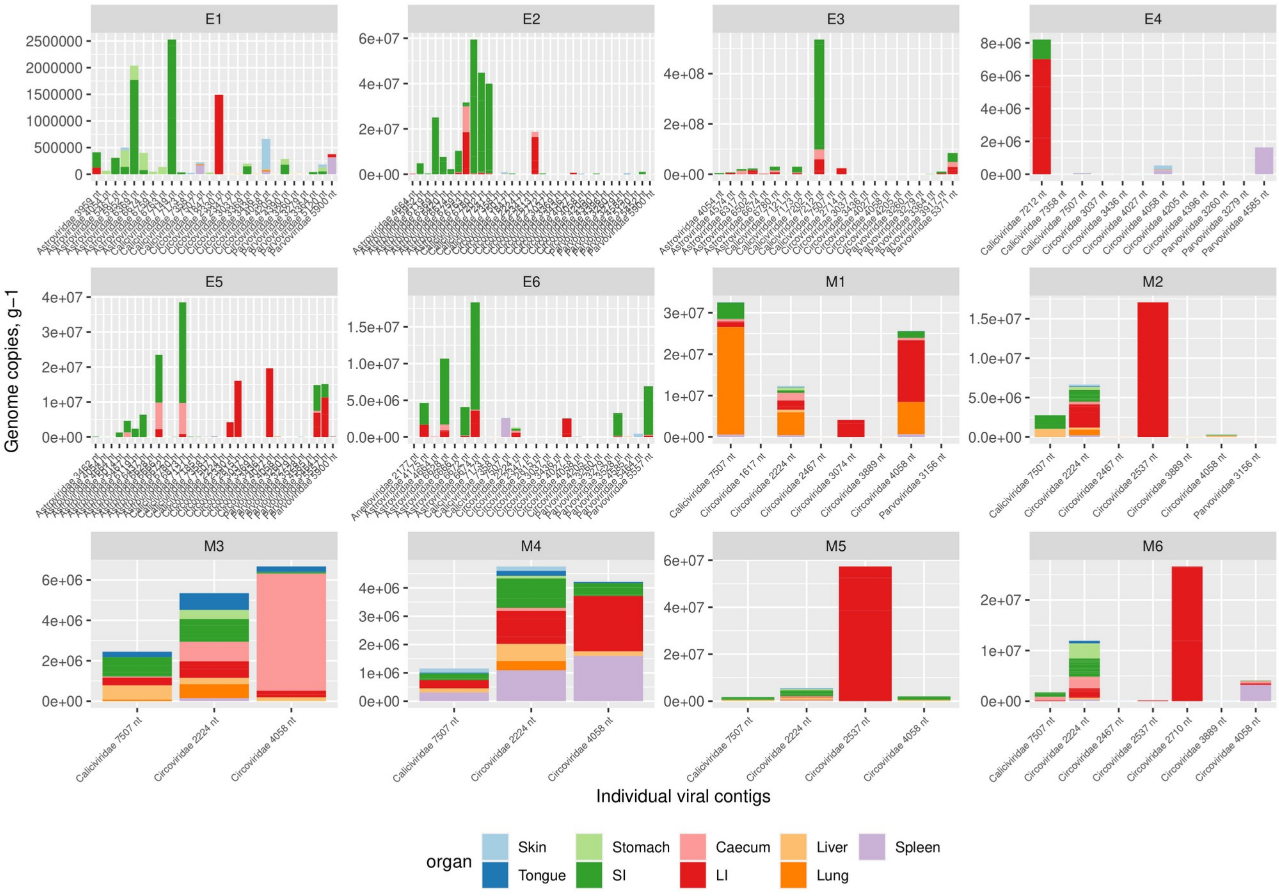

Fig. S11. Distribution of absolute abundance of individual eukaryotic viral genomic contigs across body sites in pigs and macaques. Each genomic contig (horizontal axis) in each of the animals (individual panels) roughly corresponds to a viral species. Vertical axis shows stacked absolute abundance of individual viruses across body sites (coloured bars). Each panel represent an individual animal (M1-6 for macaques, E1-6 for pigs).

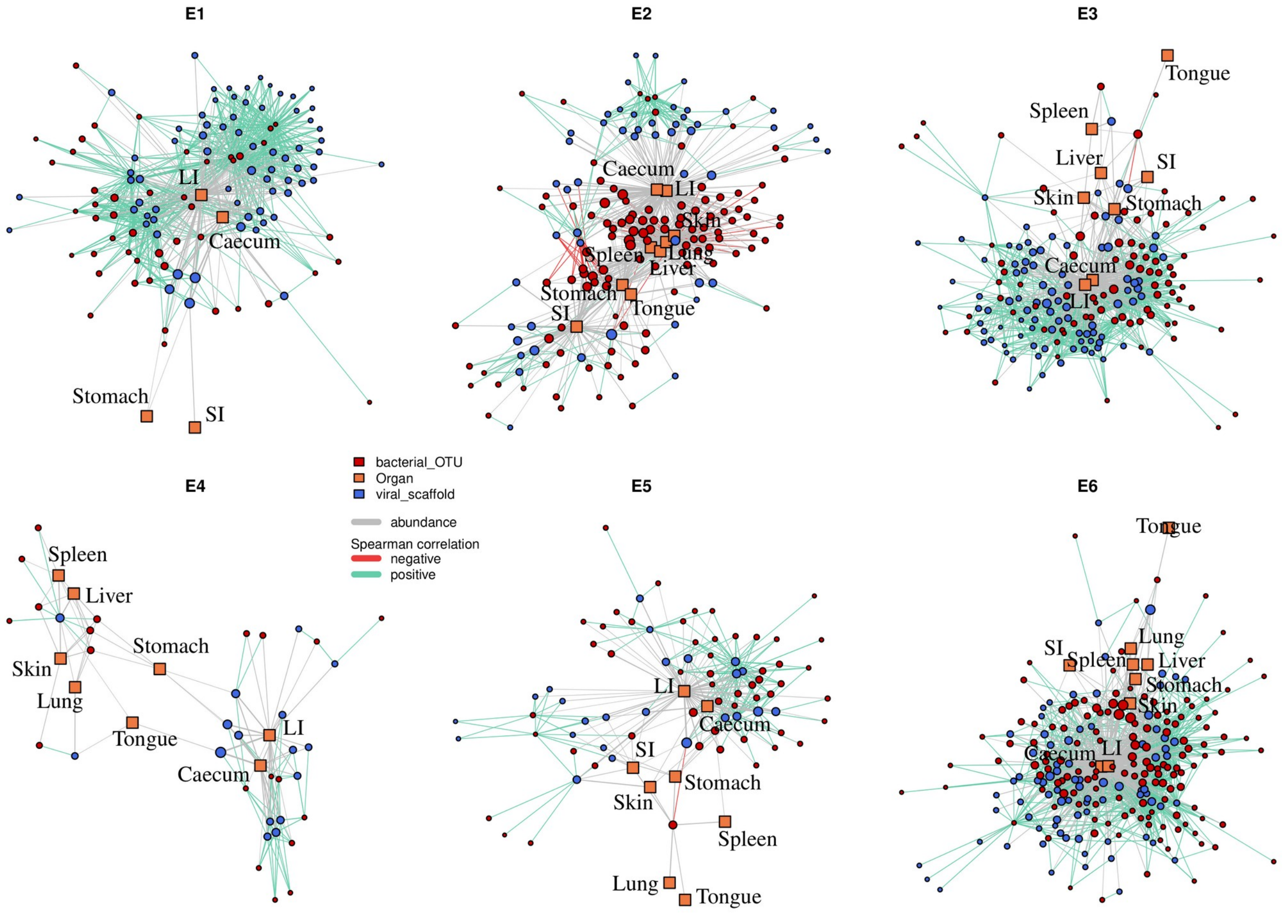

Fig. S13. Mostly positive correlation of fractional abundance between viral genomic contigs and bacterial OTUs (pigs). Networks of co-abundance between viral genomic contigs and bacterial OTUs across anatomical sites in pigs E1-E6. Network plots were created using iGraph and laid out using Fruchterman-Reingold algorithm; vertices represent organs (orange squares), bacterial OTUs (red circles), and viral genomic contigs (blue circles); grey edges thickness is proportional to log10 fractional abundance in a given organ (only cases with relative abundance of ≥ 0.1% are shown); green and red edges represent positive and negative Spearman rank correlations, respectively (between pairs of viral and bacterial OTUs; |Spearman ρ| ≥ 0.6; $p < 0.01$ in two tailed tests with Bonferroni correction).

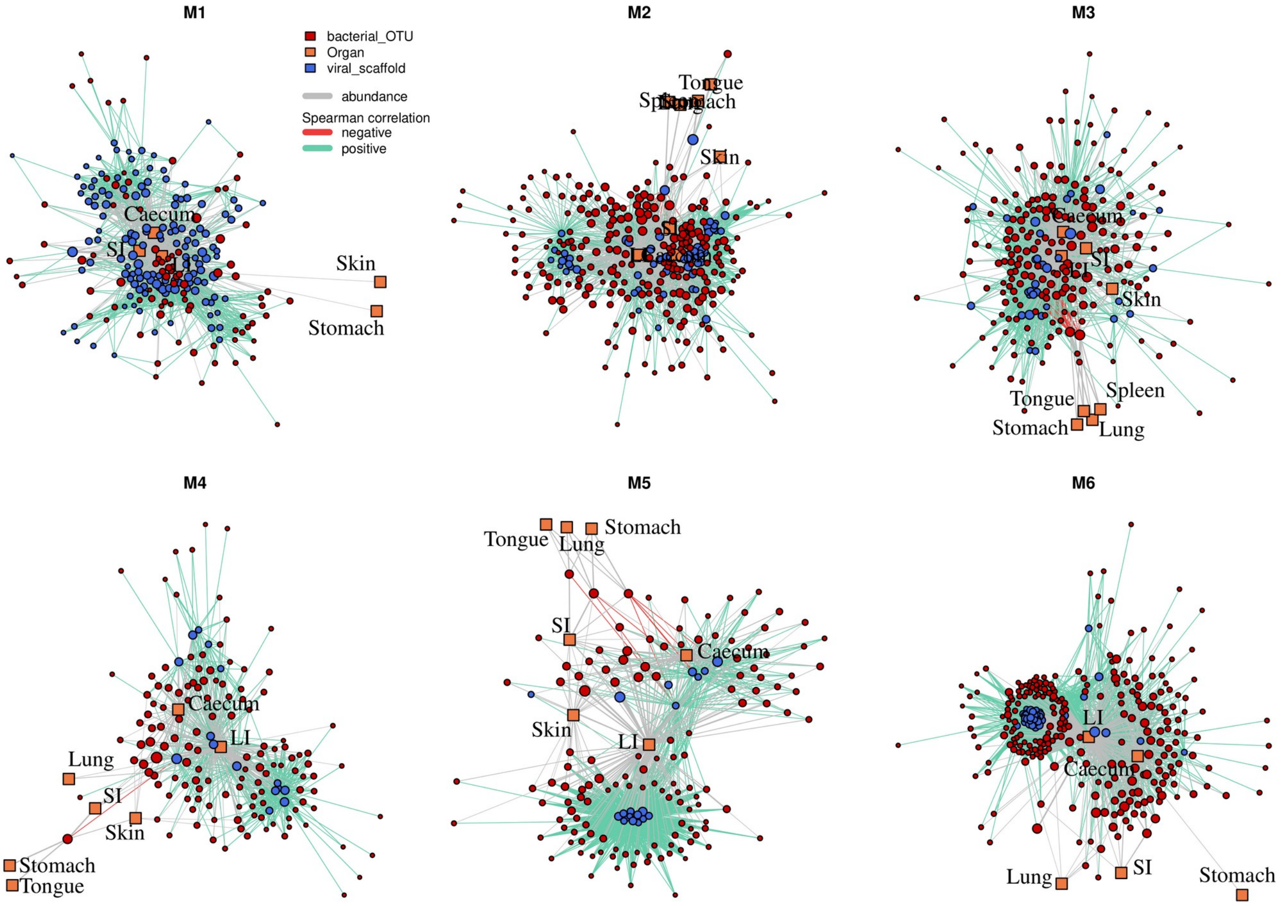

Fig. S13. Mostly positive correlation of fractional abundance between viral genomic contigs and bacterial OTUs (macaques). Networks of co-abundance between viral genomic contigs and bacterial OTUs across anatomical sites in macaques M1-M6. Network plots were created using iGraph and laid out using Fruchterman-Reingold algorithm; vertices represent organs (orange squares), bacterial OTUs (red circles), and viral genomic contigs (blue circles); grey edges thickness is proportional to log10 fractional abundance in a given organ (only cases with relative abundance of ≥ 0.1% are shown); green and red edges represent positive and negative Spearman rank correlations, respectively (between pairs of viral and bacterial OTUs; |Spearman ρ| ≥ 0.6; p < 0.01 with Bonferroni correction).

Table S1. Sample metadata table. (https://doi.org/10.6084/m9.figshare.15149247.v2**)**

References

1. Guo, J. *et al.* VirSorter2: a multi-classifier, expert-guided approach to detect diverse DNA and RNA viruses. *Microbiome* **9**, 37 (2021).
2. Devoto, A. E. *et al.* Megaphages infect Prevotella and variants are widespread in gut microbiomes. *Nature Microbiology* **4**, 693 (2019).
3. O'Leary, N. A. *et al.* Reference sequence (RefSeq) database at NCBI: current status, taxonomic expansion, and functional annotation. *Nucleic Acids Res* **44**, D733–D745 (2016).
4. Guerin, E. *et al.* Biology and Taxonomy of crAss-like Bacteriophages, the Most Abundant Virus in the Human Gut. *Cell Host & Microbe* **24**, 653-664.e6 (2018).
5. Gregory, A. C. *et al.* The Gut Virome Database Reveals Age-Dependent Patterns of Virome Diversity in the Human Gut. *Cell Host & Microbe* **28**, 724-740.e8 (2020).
6. Camarillo-Guerrero, L. F., Almeida, A., Rangel-Pineros, G., Finn, R. D. & Lawley, T. D. Massive expansion of human gut bacteriophage diversity. *Cell* **184**, 1098-1109.e9 (2021).
7. Nayfach, S. *et al.* Metagenomic compendium of 189,680 DNA viruses from the human gut microbiome. *Nat Microbiol* **6**, 960–970 (2021).
8. Roux, S. *et al.* IMG/VR v3: an integrated ecological and evolutionary framework for interrogating genomes of uncultivated viruses. *Nucleic Acids Research* **49**, D764–D775 (2021).
9. Roux, S. *et al.* Minimum Information about an Uncultivated Virus Genome (MIUViG). *Nature Biotechnology* **37**, 29–37 (2019).
10. Adriaenssens, E. M. *et al.* Taxonomy of prokaryotic viruses: 2018-2019 update from the ICTV Bacterial and Archaeal Viruses Subcommittee. *Arch Virol* **165**, 1253–1260 (2020).
11. Koonin, E. V. *et al.* Global Organization and Proposed Megataxonomy of the Virus World. *Microbiol. Mol. Biol. Rev.* **84**, (2020).
12. Koonin, E. V. & Yutin, N. The crAss-like Phage Group: How Metagenomics Reshaped the Human Virome. *Trends in Microbiology* **28**, 349–359 (2020).
13. Callanan, J. *et al.* Expansion of known ssRNA phage genomes: From tens to over a thousand. *Science Advances* **6**, eaay5981 (2020).
14. Nayfach, S. *et al.* CheckV assesses the quality and completeness of metagenome-assembled viral genomes. *Nat Biotechnol* **39**, 578–585 (2021).
15. Truong, D. T. *et al.* MetaPhlAn2 for enhanced metagenomic taxonomic profiling. *Nature Methods* **12**, 902–903 (2015).
16. Minot, S. *et al.* Rapid evolution of the human gut virome. *Proc Natl Acad Sci U S A* **110**, 12450–12455 (2013).
17. Shkoporov, A. N. *et al.* The Human Gut Virome Is Highly Diverse, Stable, and Individual Specific. *Cell Host & Microbe* **26**, 527-541.e5 (2019).
18. Manrique, P. *et al.* Healthy human gut phageome. *Proc Natl Acad Sci U S A* **113**, 10400–10405 (2016).
19. Norman, J. M. *et al.* Disease-specific Alterations in the Enteric Virome in Inflammatory Bowel Disease. *Cell* **160**, 447–460 (2015).
20. Clooney, A. G. *et al.* Whole-Virome Analysis Sheds Light on Viral Dark Matter in Inflammatory Bowel Disease. *Cell Host & Microbe* **26**, 764-778.e5 (2019).
21. Minot, S. *et al.* The human gut virome: Inter-individual variation and dynamic response to diet. *Genome Res* **21**, 1616–1625 (2011).
22. Siranosian, B. A., Tamburini, F. B., Sherlock, G. & Bhatt, A. S. Acquisition, transmission and strain diversity of human gut-colonizing crAss-like phages. *Nature Communications* **11**, 280 (2020).
23. Bin Jang, H. *et al.* Taxonomic assignment of uncultivated prokaryotic virus genomes is enabled by gene-sharing networks. *Nat Biotechnol* **37**, 632–639 (2019).
24. Yasuda, K. *et al.* Biogeography of the Intestinal Mucosal and Lumenal Microbiome in the Rhesus Macaque. *Cell Host & Microbe* **17**, 385–391 (2015).

25. Mandal, S. *et al.* Analysis of composition of microbiomes: a novel method for studying microbial composition. *Microb Ecol Health Dis* **26**, 27663 (2015).

26. Nearing, J. T. *et al.* Microbiome differential abundance methods produce different results across 38 datasets. *Nat Commun* **13**, 342 (2022).

27. Moreno-Gallego, J. L. *et al.* Virome Diversity Correlates with Intestinal Microbiome Diversity in Adult Monozygotic Twins. *Cell Host & Microbe* **25**, 261-272.e5 (2019).

28. Liang, G. *et al.* The stepwise assembly of the neonatal virome is modulated by breastfeeding. *Nature* **581**, 470–474 (2020).

29. Hockenberry, A. J. & Wilke, C. O. BACPHLIP: predicting bacteriophage lifestyle from conserved protein domains. *PeerJ* **9**, e11396 (2021).

30. Shkoporov, A. N. *et al.* Long-term persistence of crAss-like phage crAss001 is associated with phase variation in Bacteroides intestinalis. *BMC Biology* **19**, 163 (2021).

31. Silveira, C. B. & Rohwer, F. L. Piggyback-the-Winner in host-associated microbial communities. *npj Biofilms and Microbiomes* **2**, 16010 (2016).

32. Knowles, B. *et al.* Lytic to temperate switching of viral communities. *Nature* **531**, 466–470 (2016).
